# LCL161 enhances expansion and survival of engineered anti-tumor T cells but is restricted by death signaling

**DOI:** 10.3389/fimmu.2023.1179827

**Published:** 2023-04-17

**Authors:** Arya Afsahi, Christopher M. Silvestri, Allyson E. Moore, Carly F. Graham, Kaylyn Bacchiochi, Martine St-Jean, Christopher L. Baker, Robert G. Korneluk, Shawn T. Beug, Eric C. LaCasse, Jonathan L. Bramson

**Affiliations:** ^1^ Centre for Discovery in Cancer Research, McMaster University, Hamilton, ON, Canada; ^2^ McMaster Immunology Research Center, McMaster University, Hamilton, ON, Canada; ^3^ Department of Medicine, McMaster University, Hamilton, ON, Canada; ^4^ Apoptosis Research Centre, Children's Hospital of Eastern Ontario Research Institute, Ottawa, ON, Canada; ^5^ Department of Biochemistry, Microbiology and Immunology, University of Ottawa, Ottawa, ON, Canada; ^6^ Centre for Infection, Immunity and Inflammation, University of Ottawa, Ottawa, ON, Canada

**Keywords:** SMAC mimetics, engineered T cells, LCL161, multiple myeloma, cancer immunotherapy, adoptive T cell therapies

## Abstract

**Background:**

The genesis of SMAC mimetic drugs is founded on the observation that many cancers amplify IAP proteins to facilitate their survival, and therefore removal of these pathways would re-sensitize the cells towards apoptosis. It has become increasingly clear that SMAC mimetics also interface with the immune system in a modulatory manner. Suppression of IAP function by SMAC mimetics activates the non-canonical NF-κB pathway which can augment T cell function, opening the possibility of using SMAC mimetics to enhance immunotherapeutics.

**Methods:**

We have investigated the SMAC mimetic LCL161, which promotes degradation of cIAP-1 and cIAP-2, as an agent for delivering transient costimulation to engineered BMCA-specific human TAC T cells. In doing so we also sought to understand the cellular and molecular effects of LCL161 on T cell biology.

**Results:**

LCL161 activated the non-canonical NF-κB pathway and enhanced antigen-driven TAC T cell proliferation and survival. Transcriptional profiling from TAC T cells treated with LCL161 revealed differential expression of costimulatory and apoptosis-related proteins, namely CD30 and FAIM3. We hypothesized that regulation of these genes by LCL161 may influence the drug’s effects on T cells. We reversed the differential expression through genetic engineering and observed impaired costimulation by LCL161, particularly when CD30 was deleted. While LCL161 can provide a costimulatory signal to TAC T cells following exposure to isolated antigen, we did not observe a similar pattern when TAC T cells were stimulated with myeloma cells expressing the target antigen. We questioned whether FasL expression by myeloma cells may antagonize the costimulatory effects of LCL161. Fas-KO TAC T cells displayed superior expansion following antigen stimulation in the presence of LCL161, suggesting a role for Fas-related T cell death in limiting the magnitude of the T cell response to antigen in the presence of LCL161.

**Conclusions:**

Our results demonstrate that LCL161 provides costimulation to TAC T cells exposed to antigen alone, however LCL161 did not enhance TAC T cell anti-tumor function when challenged with myeloma cells and may be limited due to sensitization of T cells towards Fas-mediated apoptosis.

## Introduction

T lymphocytes have robust anti-tumor capabilities, which has established them as a leading cellular candidate for adoptive cell therapy of cancer. However, naturally occurring anti-tumor T cells are rare due to immunological tolerance mechanisms that prevent self-reactivity. Synthetic biology has overcome this issue through the development of synthetic antigen receptors that can be engineered into T cells to redirect them towards a chosen antigen. We have developed a TCR-based synthetic receptor designed to co-opt TCR signaling, known as the T cell antigen coupler (TAC), which yields potent anti-tumor T cells with reduced off-tumor toxicity (1). The activation signal transduced through the TAC receptor results in robust cytotoxicity against tumor cells, cytokine expression, and cellular proliferation.

T cell activation can be enhanced with costimulatory signaling (2). Many synthetic antigen receptors, like chimeric antigen receptors (CARs), have incorporated costimulation into the receptor for greater benefit (3, 4). However, it remains unknown whether non-canonical signaling delivered through CARs, where the TCR signaling domain is physically linked to costimulatory signaling components, contributes to the serious toxicities associated with CAR-engineered T cell therapy (5). The TAC receptor was purpose-built to deliver a canonical T cell activation signal and, hence, does not incorporate costimulatory elements into the receptor design. We recognize that provision of costimulation may enhance TAC T cell function, and thus, are pursuing chemical biology strategies to do so *via* small molecule drugs that would enable temporal and titratable costimulation without compromising the basal T cell state.

Second mitochondria-derived activator of caspases (SMAC) mimetics are relatively novel drugs under investigation as anti-cancer agents, and several have proven tolerability in clinical trials. These compounds regulate both intrinsic and extrinsic apoptosis pathways, which are dysregulated in many cancers (6, 7). SMAC mimetics have been shown to impact NF-κB signaling pathways, resulting in T cell costimulation through non-canonical NF-κB (ncNF-κB) activation (8), and subsequent increased T cell survival (9). Current data indicates that combination of SMAC mimetics with anti-tumor T cells can result in combinatorial tumor cell killing through sensitization of tumor cells to TNFα (10, 11). However, little research has been conducted on the impact of SMAC mimetics on the engineered T cell product themselves.

Here we have investigated the use of the monovalent SMAC mimetic LCL161 as an agent for delivering transient costimulation to anti-myeloma TAC T cells and to understand what subsequent changes are occurring within the T cells. We confirmed the costimulatory effects of LCL161 under conditions where TAC T cells are stimulated with antigen alone and observed enhanced survival and proliferation of T cells. However, these effects were not tied directly to the magnitude of ncNF-κB activation and we further characterized the contributions of apoptosis-related proteins, CD30, FAIM3, and Fas, to the costimulatory outcomes. Under conditions where the T cells were co-cultured with myeloma cells, we failed to see an anti-tumor benefit of LCL161 except at doses that potentiate T cell death. Deletion of Fas enhanced the survival of T cells in the presence of LCL161 and lead to enhanced expansion. Thus, although LCL161 can provide costimulation to TAC T cells, these costimulatory properties did not enhance the anti-myeloma effect of non-edited TAC T cells.

## Results

### LCL161-mediated costimulation of TAC T cells

T cell costimulation is multi-faceted and results in several downstream functional changes. Proliferative capacity and survival are two key parameters augmented by costimulation that are positively associated with clinical success of adoptive T cell therapy (12, 13), and thus were used as our primary functional readouts. To characterize the costimulatory properties of LCL161, a dye-dilution proliferation assay using CellTrace Violet was used. To determine the dose of LCL161 that provided optimal costimulation, we stimulated non-engineered T cells and engineered BCMA-specific TAC T cells (14) through the TCR or TAC receptor alone using plate-bound agonistic anti-CD3 or 6-7 µm diameter polystyrene microbeads coated with BCMA, respectively. Both non-engineered and TAC-engineered T cells demonstrated increased entry into division and increased cell yield and viability after stimulation in the presence of increasing concentrations of LCL161, which plateaued between 0.5-1 μM (**Supplemental Figure 1**). Therefore, we selected a concentration of 0.625 μM LCL161 for further experiments.

We noted donor variation in the degree of costimulation engendered by LCL161. Therefore, we generated TAC T cells from a series of healthy and myeloma donors to garner broad insights regarding the influence of donor on the biological outcomes (**Supplemental Figure 2**, **Supplemental Table 1**).

**Figure 1:**
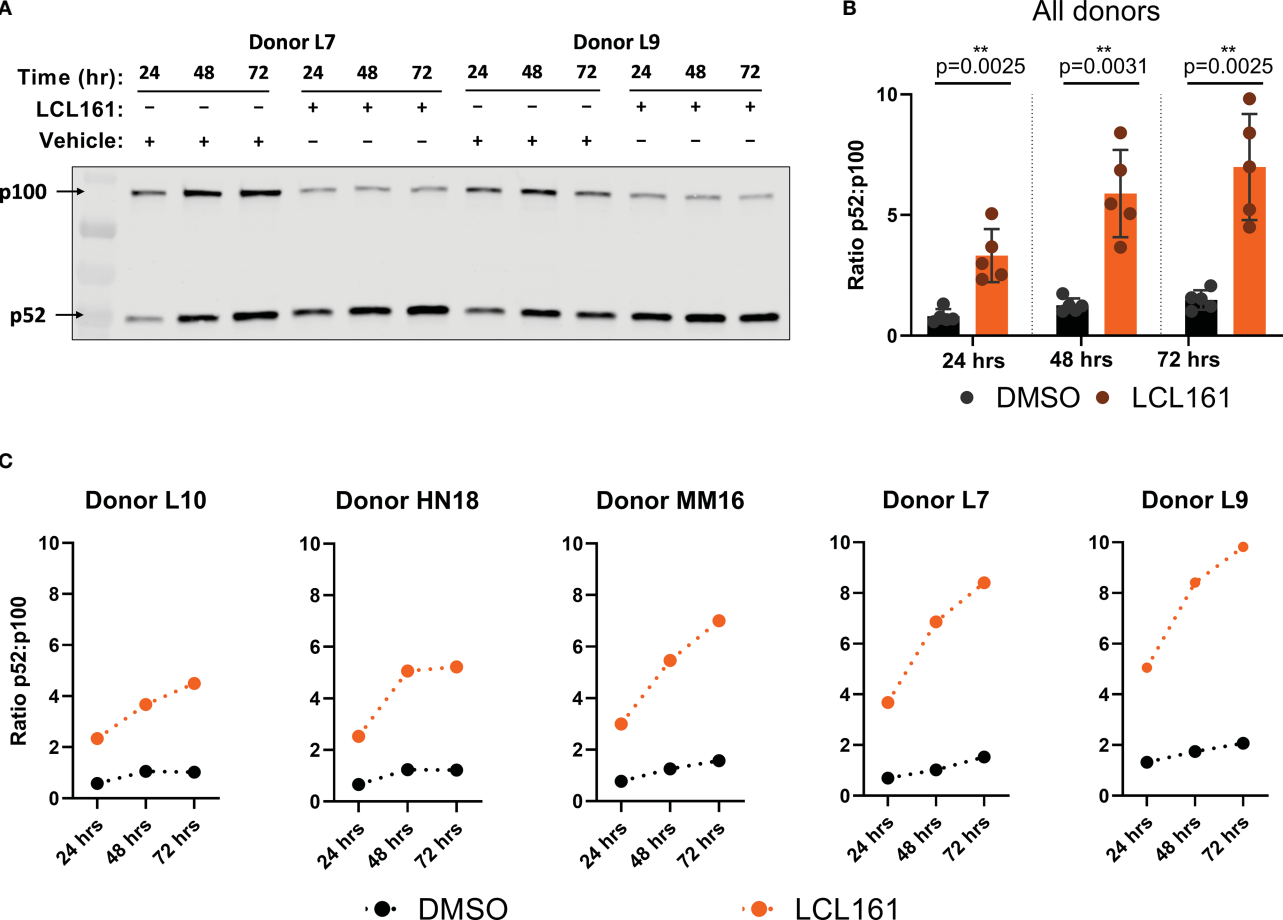
LCL161 activates the ncNF-κB signaling pathway in TAC T cells. 0.5x10^6^ TAC T cells were stimulated on plates coated with 0.05 ng/mL BCMA-Fc and either DMSO or 0.625 µM LCL161. Cells were collected and lysates were processed, ran on 4-20% polyacrylamide gels, and immunoblotted for NF-κB2 p100/p52. **(A)** Representative western blot indicating levels of NF-κB2 p100 and p52. **(B-C)** Signal quantification and normalization using Li-Cor Empiria software. NF-κB2 p100 and p52 signal was quantified and normalized to total protein loaded per lane and a ratio was calculated. **(B)** n=5. **p<0.01 as calculated by two-tailed paired t test. **(C)** data from individual donors in **(B)** These data represent 2 independent experiments.

Treatment of T cells with LCL161 should initiate ncNF-κB signaling by conversion of NF-κB2 p100 precursor to the active p52 subunit (8). TAC T cells were stimulated with plate-bound BCMA in the presence of 0.625 μM LCL161 or vehicle for 24 – 72 hours, after which the p52/p100 ratio were determined by western blot. The amount of immobilized BCMA was determined by serial dilution in a dye-dilution proliferation assay and a concentration of 0.05 µg/mL was chosen as it provided relatively weak stimulation to readily visualize LCL161 costimulation (data not shown). Stimulation with antigen and vehicle resulted in a limited increase in the p52/p100 ratio, whereas antigenic stimulation alongside LCL161 resulted in significantly greater conversion of p100 to p52 amongst all donors (**Figure 1B**) indicating activation of the signaling pathway. The relative conversion of p100 to p52 varied among the T cell products tested, indicating donor variability (**Figure 1C**).

**Figure 2:**
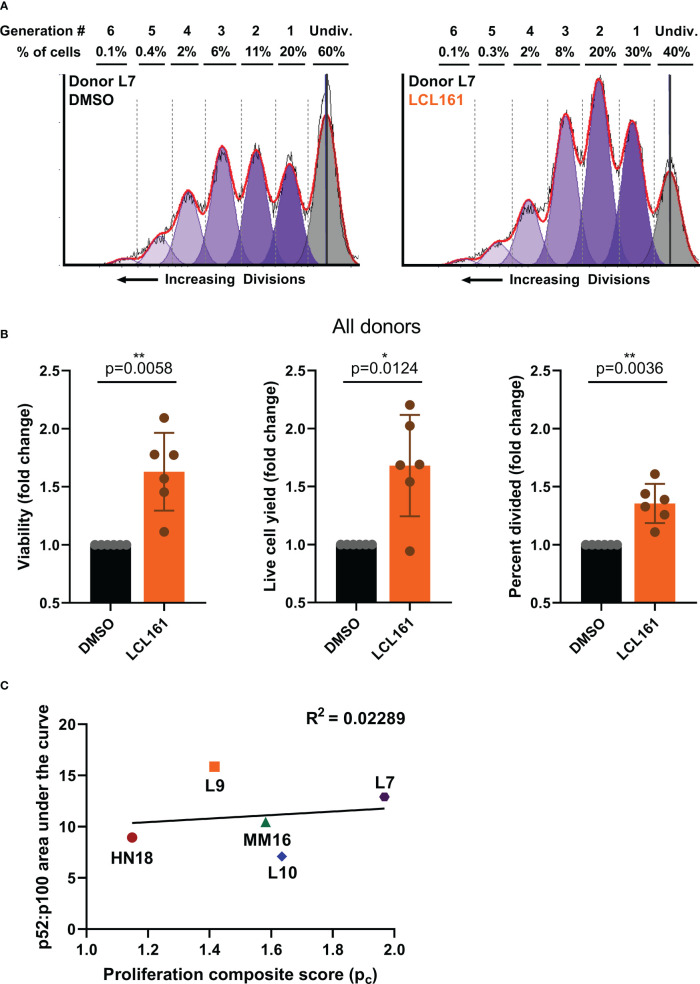
LCL161 treatment amplifies proliferation and enhances survival of antigen-stimulated TAC T cells. CTV-labelled TAC T cells were stimulated with Protein G polystyrene beads loaded with 50 ng BCMA-Fc/million beads at an E:T of 1:1 for 96 hours in the presence of 0.625 µM LCL161 or DMSO. T cells were harvested and stained for flow cytometric analysis. **(A)** representative flow plots with proliferation modeling, and **(B)** analyzed proliferation data from 5 donors, n=6; *p<0.05, **p<0.01 as calculated by two-tailed paired t test. These data represent 3 independent experiments. **(C)** Composite proliferation score was calculated as the average of the three proliferation parameters and compared to the area under the curve of the p52:p100 ratio curves in **Figure 1C**. A simple regression was calculated to determine linear fit.

Given the variability in ncNF-κB signaling when TAC T cells from different donors were stimulated with target in the presence of LCL161, we assessed the proliferation and survival of TAC T cells from the donors shown in **Figure 1**. TAC T cells stimulated with BCMA-coated beads and 0.625 μM LCL161 displayed significantly greater viability, total live cell yield, and overall percentage of cells entering division than those stimulated with beads and vehicle alone (**Figures 2A, B**). While all donors displayed some enhancement in these parameters in the presence of LCL161, the effect again varied among the donors (**Supplemental Figure 3**). We examined the relationship between p100 to p52 conversion and the proliferation metrics by creating a simple proliferation composite score (p_c_) that averages the three metrics we have examined ( $\text{pc }=\;\frac{viability+divided\%+cell\;yield}{3}$ ). We then compared this composite score to the area-under-the-curve of the p52:p100 ratio plots and found that there was no relationship between the absolute magnitude of NF-κB2 activation and the level of proliferation and survival enhancement delivered by LCL161 (**Figure 2C**, **Supplemental Figure 4**).

### CD30 and FAIM3 are implicated in the costimulatory role of LCL161

**Figure 3:**
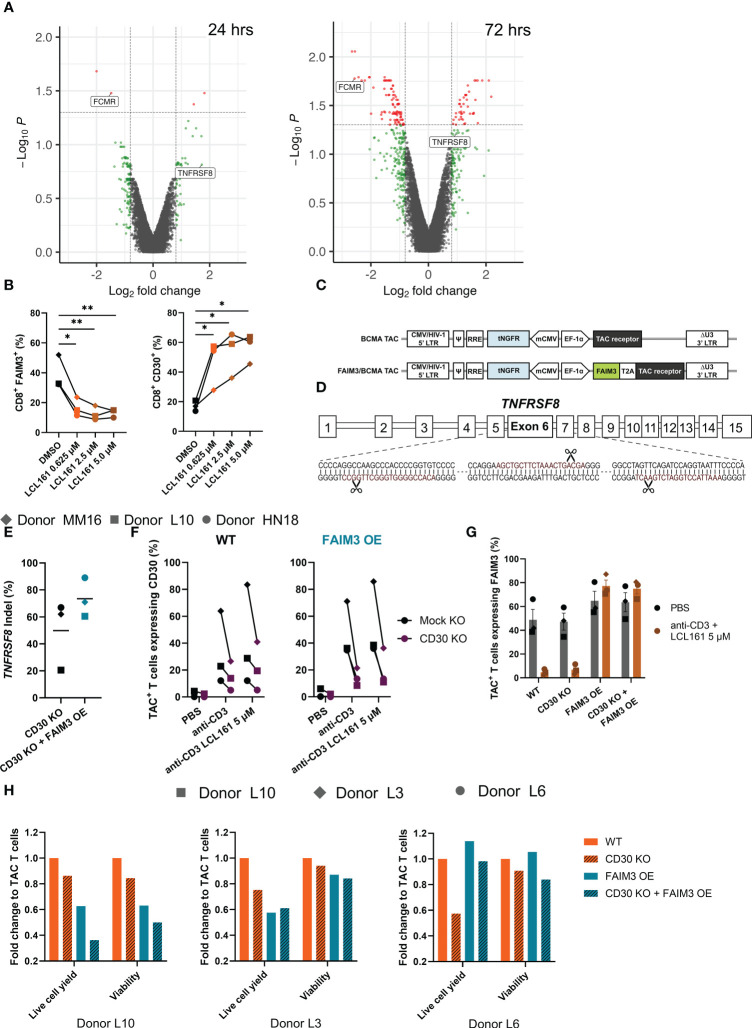
FAIM3 and CD30 mediate, in part, the costimulatory mechanisms of LCL161 in enhancing TAC T cell proliferation. **(A)** Cryopreserved TAC T cells were thawed into complete media, rested for 24 hrs, then 5x10^5^ T cells were stimulated by 0.05 μg/mL plate-bound BCMA antigen for 24 or 72 hrs in the presence of DMSO or LCL161. After stimulation, cells were collected, homogenized, and RNA was collected. Transcripts were assessed using a Clariom-S Pico human genechip. n=3, 1 per donor (HN18, L10, MM16). Volcano plots of the 24 and 72 hour Clariom data were generated using a p-value threshold of 0.05 (red dots) and fold change of 1.75 (red or green dots). **(B)** Thawed and rested cryopreserved TAC T cells were stimulated for 72 hrs with BCMA-coated microbeads and LCL161 or vehicle. Cells were collected, stained, and analyzed by flow cytometry for FAIM3 and CD30 expression. **(C)** Schematic diagram of FAIM3 cDNA separated by a T2A sequence upstream of the TAC receptor within the lentiviral vector. **(D)** *TNFRSF8* was deleted by triple gRNA editing with CRISPR/Cas9, schematic indicates the sequence of gRNA (in red) and cut sites on the targeted exon. **(E-H)** BCMA TAC T cells ± FAIM3 overexpression (OE) were cultured as normal and edited on day 3 with CD30 multi-guide or negative control gRNA/Cas9 RNP by Neon electroporation. T cells were then cultured until day 14-15 and utilized fresh in assays. **(E)** TAC T cell genomic DNA was collected on day 13-14 of culture by extraction with Lucigen DNA extraction solution. The *TNFRSF8* exon 6 locus was amplified by PCR, sequenced by sanger sequencing, and indel frequency was computed using Synthego ICE. **(F-G)** BCMA TAC T cells ± FAIM3 OE were stimulated using 1 μg/mL plate-bound agonistic anti-CD3 antibody ± 5 μM LCL161 for 24 hrs (donor L10) or 72 hrs (donors L3 and L6). After stimulation T cells were collected and stained for **(F)** CD30 and **(G)** FAIM3 expression and analyzed by flow cytometry. **(H)** CTV-labelled TAC T cells were stimulated with 0.625 μM LCL161 and BCMA-coated microbeads at an E:T 1:1 for 96 hrs then analyzed by flow cytometry. Data was modeled using FCS Express 7 and values were normalized to wildtype TAC T cells. n=3. The data in panel B represent 1 independent experiment. The data in panels E – H represent 3 independent experiments.

To gain insight into the mechanism(s) by which LCL161 influences T cell biology we performed transcriptional profiling on TAC T cells from three donors (HN18, MM16, and L10) stimulated with BCMA-coated microbeads alongside LCL161 or vehicle for 24 or 72 hrs. After stimulation, RNA was collected and analyzed using the Clariom-S Pico human genechip, which covers >20,500 genes. We primarily observed differential expression of genes related to immune function and activation (**Supplemental Figure 5**; data available at GEO, GSE227986). Of particular interest to us were two genes highly affected by LCL161, *FCMR* (encoding FAIM3) and *TNFRSF8* (encoding CD30) (**Figure 3A**). FAIM3 (15, 16) and CD30 (17–19) are both involved in regulation of costimulation and apoptosis pathways in T cells and contribute to a balance of survival and death signaling. We confirmed the transcript data by measuring the surface expression of CD30 and FAIM3 on TAC T cells following stimulation with antigen and increasing concentration of LCL161. Indeed, we observed that antigen-mediated stimulation of TAC T cells in the presence LCL161 resulted in up- and down-regulation of CD30 and FAIM3, respectively, in CD8^+^ TAC T cells (**Figure 3B**). A similar observation was seen with CD4^+^ TAC T cells (data not shown).

We sought to investigate the roles of these proteins in LCL161-mediated costimulation. We speculated that reversing the LCL161-mediated repression and induction of FAIM3 and CD30, respectively, would alter the costimulatory effects of the drug. To investigate how these receptors influence the proliferative capacity and survival of T cells during extended stimulation, we genetically engineered our BCMA TAC T cells. To force constitutive expression of *FCMR* (FAIM3 OE), we inserted a copy of the cDNA upstream of the TAC receptor in the lentiviral vector used to engineer the T cells (**Figure 3C**). To remove *TNFRSF8* expression (CD30 KO), we deleted the gene using CRISPR/Cas9 with a triple gRNA approach (**Figure 3D**). As a control, we exposed BCMA TAC T cells to a non-targeting gRNA/Cas9 RNP to account for any effect of gRNA/Cas9 or electroporation on the T cells. We used genotyping to determine CD30 gene-disruption (**Figure 3E**) and phenotypic analysis to determine CD30 absence and FAIM3 forced surface expression (**Figure 3F, G**). To do so, we stimulated TAC T cells with anti-CD3 and high dose LCL161 to maximally induce CD30 and repress FAIM3. Under the effects of LCL161 we noted a marked reduction in CD30 expression on the cell surface of the gene-edited TAC T cells compared to controls (**Figure 3F**), although we were unable to achieve complete knock down of CD30 despite extensive optimization of the knock-out protocol. Similarly, forced expression of FAIM3 was successful and surface expression was maintained in the presence of LCL161 during stimulation (**Figure 3G**). Next, we evaluated the effects of CD3O knockout and/or constitutive FAIM3 on TAC T cell proliferation and survival following stimulation with antigen-coated microbeads in the presence of LCL161. Disruption of CD30 had a profound negative impact on the proliferative capacity of TAC T cells in multiple donors, even though we did not achieve complete disruption of the CD30 gene (**Figure 3H**). The effect of FAIM3 was variable; constitutive expression of FAIM3 impaired cell expansion of TAC T cells generated from donors L10 and L3 but had no impact on TAC T cells derived from donor L6. The combination of CD30-disruption and forced expression of FAIM3 also yielded variable outcomes as the combination further impaired the cell yield from Donor L10 TAC T cells, revealed no combinatorial effect with TAC T cells from Donor L3 and, in the case of TAC T cells from Donor L6, overexpression of FAIM3 counteracted the effects of CD30 deletion. These results demonstrate that the costimulatory effects of LCL161 on TAC T cells are CD30-dependent, while the role of FAIM3 in LCL161-mediated costimulation is variable and donor-dependent.

### LCL161-mediated costimulation impairs TAC T cell expansion in co-culture with myeloma cells

**Figure 4:**
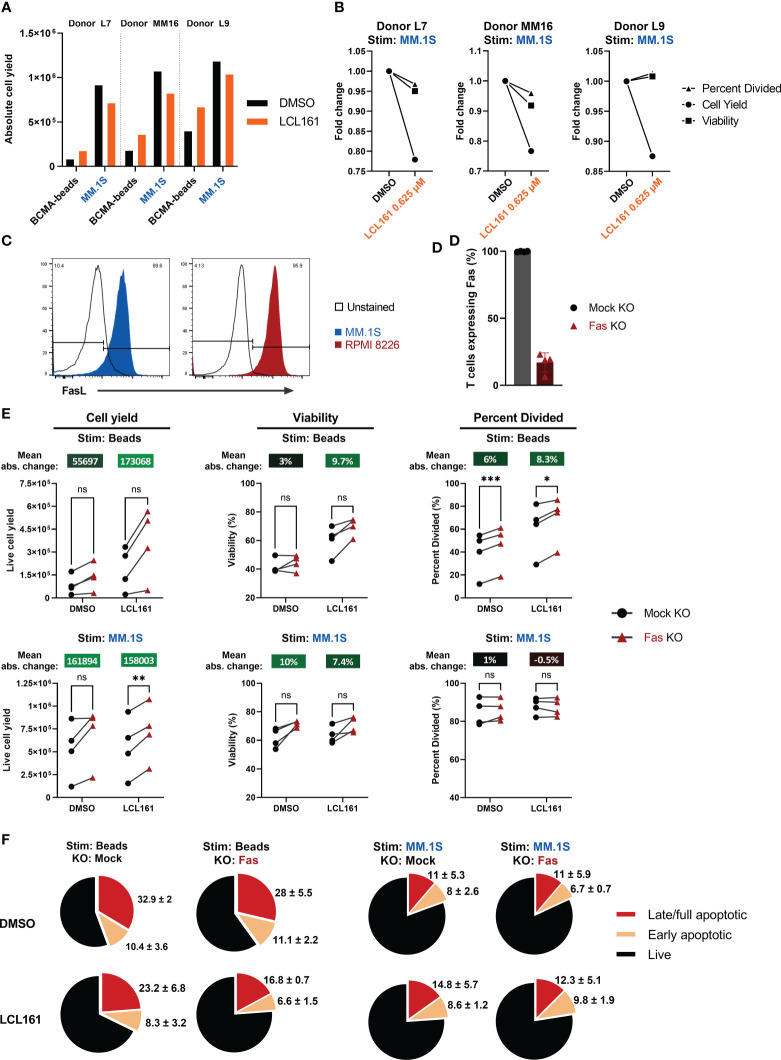
LCL161 does not enhance TAC T cell survival and proliferation when co-cultured with myeloma cells *in vitro* and is independent of Fas. **(A-B)** Thawed and rested cryopreserved BCMA TAC T cells were stimulated 1:1 with MM.1S tumor cells or BCMA-coated microbeads in the presence of 0.625 μM LCL161 for 96 hrs. Absolute cell count was enumerated using counting beads during flow cytometric analysis. The bead proliferation data displayed in **(A)** is shared in **Figure 2B**. **(B)** Proliferation metrics from TAC T cells stimulated with MM.1S cells were normalized to the vehicle control. **(C)** Cultured MM.1S and RPMI 8226 myeloma cells were stained for FasL expression and analyzed by flow cytometry. **(D–F)** BCMA TAC T cells were cultured as normal and edited on day 3 with Fas multi-guide or negative control gRNA/Cas9 RNP by Neon electroporation. T cells were then cultured until day 14-15 and utilized fresh in assays. n=4. **(D)** BCMA TAC T cells edited with mock or Fas RNP were stained for Fas expression on day 13-14 and analyzed by flow cytometry. **(E)** Mock or Fas-edited TAC T cells were stimulated 1:1 with MM.1S tumor cells or BCMA-coated microbeads and in the presence of 0.625 μM LCL161 or vehicle for 96 hrs. After stimulation, samples were stained, mixed with counting beads, and analyzed by flow cytometry. Proliferation metrics were modeled using FCS Express 7 software. Mean absolute change between mock and Fas KO is reported above each condition. **(F)** Mock or Fas-edited TAC T cells were stimulated 1:1 with MM.1S tumor cells or BCMA-coated microbeads and in the presence of 0.625 μM LCL161 or vehicle for 72 hrs. After stimulation, cells were stained for live/dead, fixed, and finally stained for phosphatidylserine (PS) on the outer membrane. Cells were analyzed by flow cytometry. Early apoptotic cells were defined as live/dead^-^, PS^+^, late/full apoptotic cells were defined as live/dead^+^, PS^+^. Pie charts represent mean values, with mean + standard deviation written outside the pie slice. n=3. These data represent 3 independent experiments. ns: not significant, *p<0.05, **p<0.01, and ***p<0.001 as calculated by paired t test with Holm-Šídák method.

To assess whether LCL161 could provide costimulatory benefit when TAC T cells were co-cultured with myeloma cells, we stimulated T cells with BCMA^+^ MM.1S myeloma tumor cells in the presence or absence of 0.625 µM LCL161. The absolute proliferative response of the TAC T cells to the myeloma cell line was much more robust than the antigen-coated beads (**Figure 4A**; data from **Figure 2** is included as a point of reference). Although MM.1S stimulation gave an overall stronger proliferative signal, the combination of LCL161 and MM.1S stimulation resulted in reduced expansion of the TAC T cells, which manifest as a decrease in TAC T cell numbers at the end of the co-culture, and there was no augmentation of survival or cells entering division as previously observed with bead stimulation (**Figure 4B**). These data reveal that while LCL161 costimulation was important when TAC T cells were stimulated with antigen alone, there may be opposing signals delivered by the tumor cells which mitigate the beneficial impacts of the LCL161.

T cell expansion can be limited by Fas-mediated death (20), and the reduced apoptosis threshold as a result of LCL161 targeting of IAPs may underpin the negative effects of LCL161 in the context of the T cell:myeloma cell cultures. Indeed, we confirmed that the tumor lines used in this study uniformly expressed FasL (**Figure 4C**). To address the possibility that Fas signaling mitigates the beneficial effects of LCL161, we removed Fas from the TAC T cells by CRISPR-mediated gene-editing. Here, we reproducibly achieved a high efficiency KO at >80% in all cases (**Figure 4D**). The removal of Fas in the TAC T cells enhanced total cell yield, viability, and cells entering division of TAC T cells in the presence of 0.625 µM LCL161 when T cells were stimulated with antigen alone (“Beads”; **Figure 4E**). This effect was greater in cells treated with LCL161 rather than DMSO. Furthermore, we observed that the incidence of apoptotic T cells was reduced following stimulation with antigen alone in the presence of LCL161 when Fas was disrupted, supporting a role for Fas in limiting the costimulatory effects of LCL161, presumably through fratricide (“Beads”; **Figure 4F**). Removal of Fas from the TAC T cells did improve cell yield following stimulation with MM.1S, but did not impact viability, cell division or incidence of apoptosis in LCL161 treated T cells compared to DMSO (“MM.1S”; **Figures 4E–F**).

### LCL161 enhancement of TAC T cell anti-tumor cytotoxicity is accompanied by T cell toxicity

**Figure 5:**
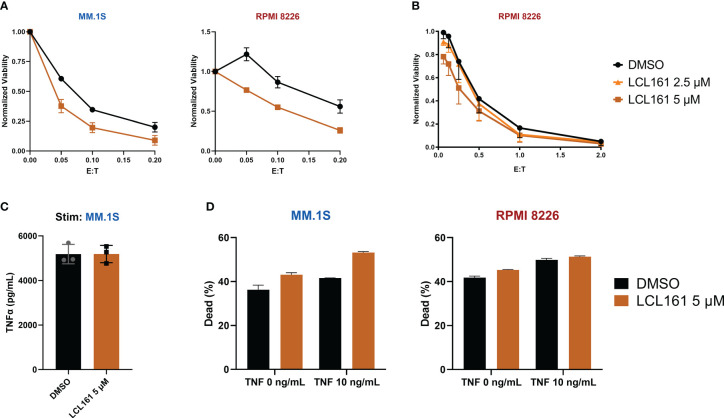
LCL161 sensitizes multiple myeloma cells to T cell cytotoxicity at higher doses. **(A)** 5x10^5^ tumor cells were co-cultured with TAC T cells and 5 μM LCL161 or vehicle, in biological triplicate at various E:T for 24 hrs. After co-culture, cytotoxicity was measured through flow analysis and normalized to tumor-only controls; data shown as mean and SD. Data were generated with T cells from 3 different donors (L5, L6, L9) run independently in 1 experiment **(B)**. Luciferase-based cytotoxicity assay of TAC T cells co-cultured with effLuc+ myeloma cell lines for 48 hours in the presence of vehicle or LCL161. Samples were run in triplicate and normalized to tumor-only cell controls; data shown as mean and SEM. Data were generated with T cells from 3 different donors (L6, L7, L9) run independently in 1 experiment. **(C)** TAC T cells were stimulated with MM.1S cells at an E:T ratio of 1:1 in the presence of either DMSO or LCL161 for 24 hours and TNFα levels were measured in the supernatant. The data reflects technical triplicates **(D)** Multiple myeloma cell lines were incubated with DMSO or LCL161, in the presence of TNFα for 48 hrs. Cells were harvested, stained for viability, and analyzed by flow cytometry. The data reflects technical triplicates.

Although we did not observe enhanced proliferation or survival on TAC T cells stimulated with myeloma tumor cell lines *in vitro*, we wondered if LCL161 would augment T cell-mediated cytotoxicity against myeloma cells. To this end, TAC T cells were co-cultured with myeloma cells in the presence of LCL161 and tumor viability was assessed. Enhanced killing of the tumor cells was only observed with high concentrations of LCL161 tested (5 μM; **Figure 5A**). In fact, a small change in concentration to 2.5 μM failed to provide enhanced killing (**Figure 5B**). Inhibition of the IAPs in tumor cells is predicted to result in increased sensitivity to TNF-mediated cell death by switching TNFα signaling from activation of the classical NF-κB pathway towards caspase-8 mediated apoptosis (21, 22), and we have observed that TAC T cell produce large amounts of TNFα upon stimulation by myeloma cell lines (**Figure 5C**). To address whether LCL161 augments TAC T cell killing through sensitization of the multiple myeloma tumor cells to TNFα, we cultured MM.1S or RPMI 8226 cell lines with 5 μM LCL161 +/- recombinant human TNF. MM.1S cells displayed a modest increase to cell death when treated with both LCL161 and TNF, whereas RPMI 8226 cells remained largely unaffected (**Figure 5D**). This was not entirely unexpected as there is considerable heterogeneity in susceptibility of MM cell lines to LCL161 (23), which may be in part be due to deletions of cIAP1/2 or other NF-κB-associated factors that occur in some multiple myelomas (24, 25). Investigating these two cell lines further showed a complete basal absence of cIAP2 and p100 in the RPMI 8226 cells (**Supplemental Figure 6**), which may explain the lack of sensitization to TNFα. Thus, the increased killing of the myeloma cell lines in the presence of higher concentration of LCL161 (5 μM) is likely due to something other than sensitization of TNFα-mediated killing.

**Figure 6:**
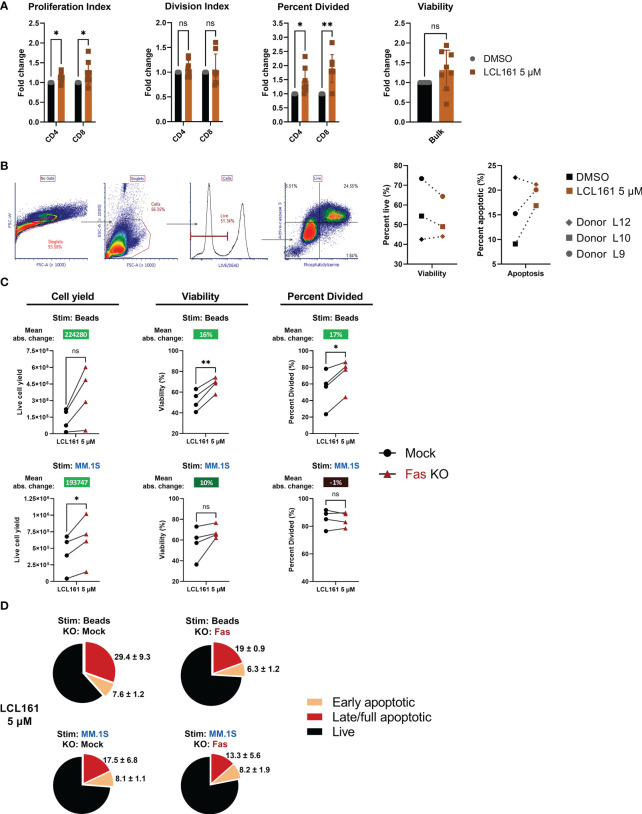
High dose LCL161 initiates activation induced cell death in stimulated TAC T cells that is responsive to Fas KO only in the case of antigen-alone stimulation. **(A)** TAC T cells labeled with CellTrace Violet were cultured with loaded beads at an E:T of 1:1 for 96 hrs. T cells were harvested and stained for flow cytometric analysis. Proliferation metrics were modeled with FCS Express 7. Proliferation and division indices are a measure of the average number of cells any or a dividing cell generates, respectively. Paired and unpaired parametric t tests with Holm-Šídák correction was used to calculate statistical significance; ns, P>0.05, *P<=0.05, **P<= 0.01. n=8, comprising 5 different donors generated in 7 independent experiments **(B)** TAC T cells were stimulated with protein G polystyrene beads coated with antigen-loaded microbeads for 48 hrs. Viability and proportion of apoptotic cells as defined by live/dead, phosphatidylserine and active-caspase 3 staining. Viable cells are defined as live/dead negative, and apoptotic cells defined as active caspase3^+^ and PS^+^. Data represent T cell products from 3 donors produced in 3 independent experiments. **(C)** CTV-labeled mock or Fas-edited TAC T cells stimulated 1:1 with MM.1S tumor cells or BCMA-coated microbeads and in the presence of 5 μM LCL161 or vehicle for 96 hrs. After stimulation, samples were stained, mixed with counting beads, and analyzed by flow cytometry. Proliferation metrics were modeled using FCS Express 7 software. Mean absolute change between mock and Fas KO is reported above each condition. Data were generated with T cells from 4 donors (L15, M12, M28, M46) produced in 3 independent experiments. **(D)** Mock or Fas-edited TAC T cells were stimulated 1:1 with MM.1S tumor cells or BCMA-coated microbeads and in the presence of 5 μM LCL161 or vehicle for 72 hrs. After stimulation, cells were stained for live/dead, fixed, and finally stained for phosphatidylserine. Cells were analyzed by flow cytometry. Early apoptotic cells were defined as live/dead^-^, PS^+^, late/full apoptotic cells were defined as live/dead^+^, PS^+^. Pie charts represent mean values, with mean + SD written outside the pie slice. Data were generated with T cells from 3 donors (M12, M28, M46) produced in 2 independent experiments.

The results in **Figure 2** indicated that maximal costimulation of the T cells is achieved with an LCL161 concentration below 1 μM, however the results in **Figure 5** indicated that concentrations of 5 µM were required to observe enhanced killing of myeloma tumor cells. We, therefore, explored the effect of 5 µM LCL161 on TAC T cells. While we observed that this high concentration of LCL161 could significantly enhance the percent of T cells that entered division (**Figure 6A**), we also noted a concomitant increase in activation induced cell death (AICD). We excluded strictly dead/necrotic cells and measured the portion of live TAC T cells that were sensitized by 5 μM LCL161 towards apoptosis by measuring active caspase-3 expression and the presence of phosphatidylserine on the outer membrane leaflet of TAC T cells stimulated with antigen-loaded beads for 48 hrs. We observed a trend towards increased proportion of stimulated TAC T cells entering apoptosis and decreased viability (**Figure 6B**). We questioned whether this observation might be explained by a second IAP target of LCL161, X-linked IAP (XIAP). As XIAP is a regulator of caspase-3, -7, and -9 related apoptotic pathways (26), depression of caspase-inhibition by XIAP at high concentrations of LCL161 may further potentiate T cells towards death (27). We stimulated T cells from two healthy donors (M12 and L15) with plate-bound BCMA-Fc and 5 μM LCL161 for 72 hours and examined the levels of XIAP within these cells, but we did not observe any difference in XIAP levels (**Supplemental Figure 7**). We questioned if the impact of Fas is more pronounced with high dose LCL161 due to sensitization of TAC T cells towards death at the 5 μM dose. Fas KO-TAC T cells were stimulated with antigen-coated beads or MM.1S myeloma cells and we assessed proliferative capacity, viability, and susceptibility to apoptosis in the presence of 5 μM LCL161 (**Figures 6C–D**). The deletion of Fas again manifested greater enhancement to proliferation and viability, and reduction to apoptosis in bead-stimulated T cells than MM.1S stimulated T cells. The mean absolute change in proliferation metrics and viability of Fas-deleted TAC T cells stimulated with bead-based antigen in the presence of 5 μM LCL161 was greater than previously measured at the lower 0.625 μM dose, suggesting that Fas limits T cell expansion under these conditions. Similar to the observation with 0.625 μM LCL161, TAC T cells stimulated with myeloma cells in the presence of 5 μM displayed enhanced accumulation of TAC T cell at the end of the culture period but no significant improvement to viability, which points to a mechanism unrelated to Fas.

## Discussion

**Figure 7:**
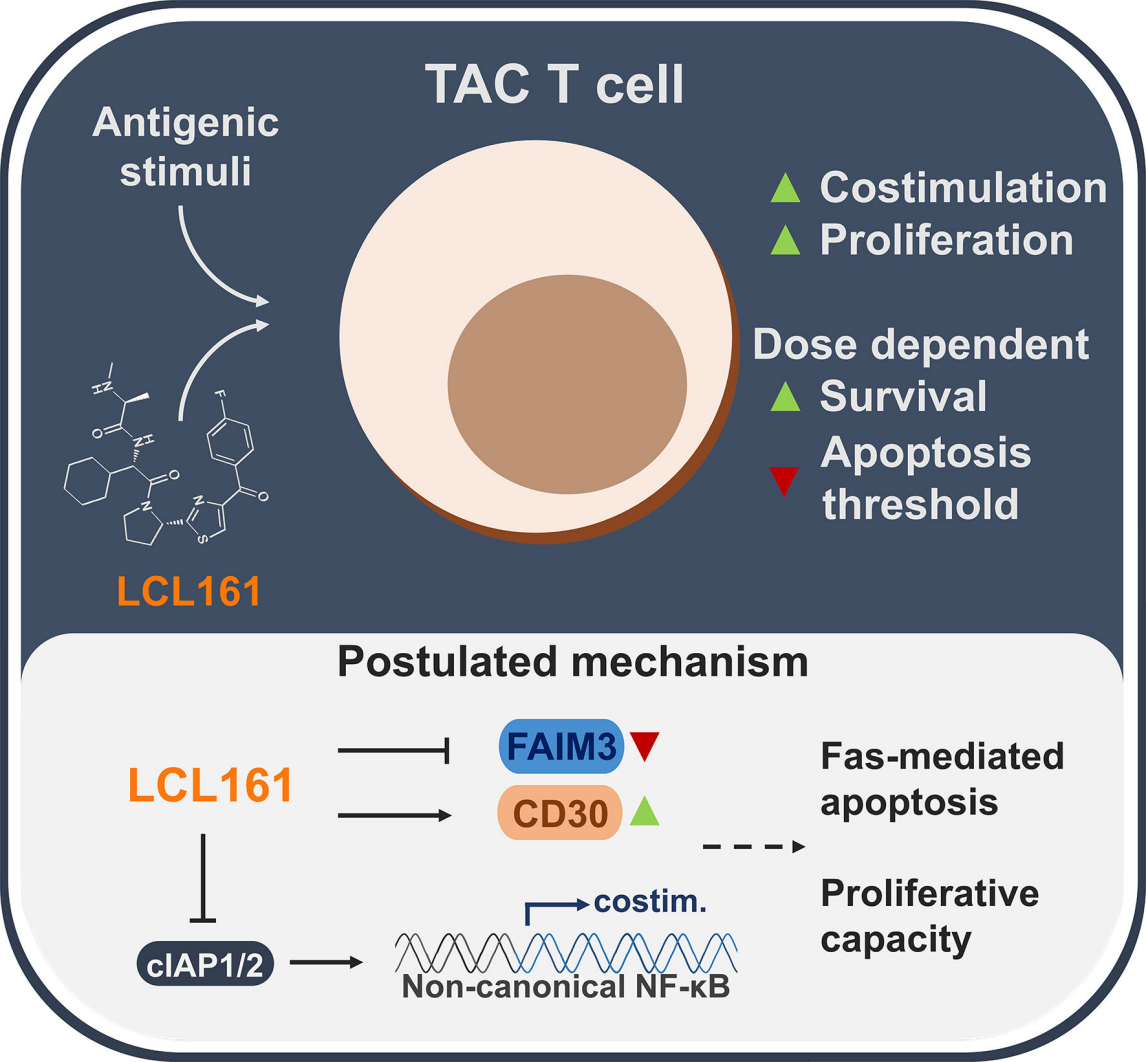
A summarized overview of the observations and postulated mechanisms of LCL161-effects on TAC T cell biology.

Unlike chimeric antigen receptors which require the inclusion of costimulation domains for optimal function (3), TAC receptors provide robust anti-tumor immunity without the need for engineered costimulation. Indeed, other synthetic receptor strategies that utilize TCR-centric designs also show potent function in the absence of costimulation (28, 29). These properties notwithstanding, costimulation promotes survival, greater proliferative capacity (30), and memory formation/persistence (31) – important traits for anti-tumor engineered T cell products (32). Herein we investigated a strategy for controlled and transient druggable delivery of costimulation using the clinical candidate, LCL161. We observed an enhancement to TAC T cell survival and proliferative capacity that was restricted at higher doses of LCL161, partially through a Fas-mediated mechanism (**Figure 7**).

Our study employed engineered TAC T cells from both healthy donors and multiple myeloma patient donors to broaden the applicability and relevance of our findings. A reductionist antigen-on-bead system allowed us to fine tune signaling events and stringently study the effects of LCL161 on our engineered TAC T cells. Costimulation from IAP antagonism is mediated through the ncNF-κB pathway (8), an observation we corroborated. Low dose LCL161 potentiated processing of NF-κB2 p100 to p52 that increased for at least 72 hrs following TAC-mediated stimulation. Functionally, low dose LCL161 provided functional improvements to T cells measured by enhanced survival and expansion. We noted that the extent to which the LCL161 enhanced survival and expansion did not appear to correlate with the ratio of p100 to p52 conversion; for example, donor HN18 displayed little enhancement of proliferation with LCL161 yet strongly activated the ncNF-κB pathway. While the exact mechanism underpinning this donor-to-donor variability remains unclear, genechip transcriptional profiling identified numerous targets which led us to investigate in more detail the roles of CD30 and FAIM3 in LCL161-mediated enhancement of TAC T cell proliferation. How these receptors function is not fully elucidated within the field of T cell biology; research is mixed with some studies showing a pro-apoptotic capacity (17, 18), and others showing a costimulatory or survival property (15, 16, 19). CD30 is a well-known TNFRSF member that plays a pivotal role in T cell activation as well as providing survival signals for lymphomas that is cIAP-dependent (33). Indeed, SMAC mimetic treatment has been shown to provide similar cellular signals as CD30, intersecting on canonical and ncNF-κB signaling (34, 35). Our data with CD30 KO clearly demonstrated its requirement in enhancing proliferation and survival in the context of LCL161 treatment. The impact of FAIM3 overexpression was less clear due to donor variation, but the data indicated a trend towards restricting proliferation when it is constitutively expressed in TAC T cells. A recent report suggested that costimulation *via* FAIM3 was dependent on the availability of its ligand, IgM (16). We reasoned that bovine IgM present in our culture medium may not provide an appropriate ligand, so we added exogenous human IgM to our culture medium but found no additive effects with the FAIM3 overexpressing cells (data not shown).

Although we observed a slight enhancement to TAC T cell-mediated killing of multiple myeloma cells *in vitro* at high doses of LCL161 (5 μM), this effect did not persist with a 2-fold reduction in concentration. Indeed, we expanded our investigation to *in vivo* studies and similarly observed no benefit in anti-tumor activity of TAC T cells against system MM.1S xenografts (described in our recent publication (14)) when mice were concomitantly treated with either 25 or 75 mg/kg LCL161 (data not shown). Our data indicate that the negative effects of the drug on the T cell population need to be carefully considered. A balance between the costimulatory effects of LCL161 and unwanted induction of AICD in the T cells became evident as we titrated the dose of drug on activated T cells, an observation previously seen with non-engineered T cells at high doses of LCL161 (36). We suspected that knockdown of both cIAP1/2 and XIAP at higher doses of LCL161 could render the T cells more susceptible to full caspase-3 activation and subsequent cell death (27). This may be exacerbated by TNFα produced by the TAC T cells, which can further potentiate death signaling in the absence of cIAP1/2 (22, 37). We did not observe increased knockdown of XIAP at increasing LCL161 doses 72 hrs after antigenic stimulation. However, it is possible that XIAP is competitively inhibited rather than degraded, and thus is not suppressing caspase-activation or death signaling. Indeed, the apoptotic sensitization in the T cells may be transduced through death-receptor pathways as XIAP is known to restrict Fas-mediated apoptosis (38). As activated T cells express Fas and FasL for regulation and immune homeostasis (39), we speculated that expression of FasL on the tumor cells contributed to the restricted ability of LCL161 to enhance TAC T cell survival and proliferation in the context of myeloma cell stimulation. In addition to this, T cell fratricide through Fas-FasL interactions with neighbouring T cells would compound this effect. To address this, we knocked out expression of Fas on our TAC T cells and showed a reduction to T cell toxicity at the low and high doses of LCL161 with bead stimulation, supporting our hypothesis that LCL161 does reduce the threshold to apoptosis in activated T cells through Fas-driven fratricide. However, this effect did not translate as clearly to MM.1S cell-based stimulation which indicates that other factors or mediators are likely involved in the restriction of LCL161-mediated proliferative enhancement with myeloma cell lines. Fas-deletion recovers the loss of TAC T cell yield observed following MM.1S stimulation in the presence of 5 μM LCL161, which supports the hypothesis that the decline in T cell yield following stimulation with MM.1S is mediated, at least in part, through Fas signaling. Nevertheless, even in the absence of Fas, we do not observe an increase in TAC T cell viability or proliferation in the presence of LCL161, which suggests that LCL161-mediated costimulation is inconsequential in the presence of the full complement of adhesion molecules and costimulatory ligands (such as LFA-3 [40)] found on the myeloma cells. Indeed, we have detected CD137L and CD86 on RPMI 8226 and MM.1S cells by flow cytometry (data not shown).

Given that the myeloma lines RPMI 8226 and MM1S were resistant to LCL161 (23) and TNFα, we were able to tune our investigations to draw conclusions under the lens of a TAC T cell-centric observation and understand the impact on T cell biology. Our study highlights several pathways and mechanisms by which LCL161 integrates into T cell proliferation and survival, expanding our understanding of SMAC mimetics as enhancers of anti-cancer immunity (41). Additional refinement to SMAC mimetic small molecule drugs through medicinal chemistry, or development of direct cIAP1/2 antagonists, could increase the therapeutic threshold between costimulatory effects and unwanted sensitization to AICD. Beyond their use as drugs to activate costimulation in the context of cell therapy, the evidence that LCL161 can enhance T cell proliferation *ex vivo* in response to a reductionist stimulation suggests that small-molecule IAP antagonists may be of use during the manufacturing of engineered T cells through enhancement of proliferation.

## Methods

### Cell lines

Human myeloma cell lines RPMI-8226 (CCL-115; purchased from ATCC (Manassas, VA) and MM.1S (kindly provided by Dr. Kelvin Lee, Roswell Park Cancer Institute, NY) were cultured in RPMI 1640 (Gibco (Thermo Fisher Scientific), Waltham, MA), supplemented with 10% FBS (Gibco), 2 mM L-glutamine (BioShop Canada Inc., Burlington, ON, Canada), 10 mM HEPES (Roche Diagnostics, Laval, QC, Canada), 1 mM sodium pyruvate (Sigma-Aldrich Canada Co. (Millipore Sigma), Oakville, ON, Canada), 1 mM nonessential amino acids (Gibco), 100 U/mL penicillin (Gibco), and 100 µg/mL streptomycin (Gibco). To generate luciferase-expressing cell lines, parental RPMI 8226 or MM.1S cell lines were transduced with lentivirus encoding enhanced firefly luciferase (effLuc) (42) as well as puromycin N-acetyltransferase at an MOI 10 and selected in culture media supplemented with 8 µg/mL puromycin (InvivoGen, San Diego, CA). HEK293T cells (kindly provided by Dr. Megan Levings, University of British Columbia, Canada) were cultured in DMEM (Gibco), supplemented with 10% FBS (Gibco), 2 mM L-glutamine (BioShop), 10 mM HEPES (Roche Diagnostics), 100 U/mL penicillin (Gibco), and 100 µg/mL streptomycin (Gibco), or 0.1 mg/mL normocin (InvivoGen). All cells were cultured at 37°C, 95% ambient air, 5% CO2, and routinely tested for mycoplasma contamination using the PlasmoTest detection kit (InvivoGen, San Diego, CA, USA).

### Lentivirus production

Lentivirus encoding the BCMA-specific TAC receptor and truncated NGFR transduction marker (14) with or without FAIM3 was produced as previously described (43) using a third generation packaging system (44). Briefly, 12 x 10^6^ HEK293T cells plated on a 15-cm dish (NUNC (Thermo Fisher Scientific)) were transfected with plasmids pRSV-REV (6.25 μg), pMD2.G (9 μg), pMDLg-pRRE (12.5 μg), and pCCL BCMA TAC (32 μg) encapsulated with Lipofectamine 2000 (Thermo Fisher Scientific) in Opti-MEM media (Gibco). 12-16 hours post-transfection, media was exchanged with HEK293 media supplemented with 1 mM sodium butyrate (Sigma-Aldrich). 24-36 hours later, supernatant was harvested, filtered through a 0.45 μm asymmetric polyethersulfone filter (Thermo Fisher Scientific) to remove cellular debris. Viral particles were then concentrated using an Amicon Ultra 15 100 kDa centrifugal filter (Millipore Sigma) and stored at -80°C. Viral titre (TU/mL) was determined post-thaw by serial dilution and transduction of HEK293T cells, and enumeration of percent NGFR^+^ cells by flow cytometry.

### Generation of engineered human T cells

Peripheral blood mononuclear cells (PBMCs) were obtained from healthy or myeloma patient donors who provided informed written consent in accordance with the Hamilton Integrated Research Ethics Board, or were collected from commercial leukapheresis products (HemaCare, Northridge, CA and STEMCELL Technologies, Vancouver, BC, Canada). In the case of leukapheresis products, samples were transported at room temperature and processed within 24 hrs of collection. Whole blood was collected from donors using BD CPT sodium heparin collection tubes (BD Biosciences). PBMCs were isolated from blood or leukapheresis by Ficoll-Paque Plus gradient centrifugation (Cytiva, Vancouver, BC, Canada) and cryopreserved in inactivated human AB serum (Corning, Corning, NY) containing 10% DMSO (Sigma-Aldrich), or in CryoStor10 (STEMCELL Technologies) for healthy donors, and RPMI (Gibco) containing 12.5% human serum albumin (Sigma-Aldrich) and 10% DMSO (Sigma-Aldrich) for myeloma patient donors. Samples were cryopreserved in an isopropanol controlled-rate freezer (Thermo Fisher Scientific) at -80°C for 24-72 hrs prior to long term storage in liquid nitrogen. Post-thaw, PBMCs were activated with 25 μL ImmunoCult soluble anti-CD3/28/2 tetrameric complexes (STEMCELL Technologies) per mL PBMC, and cultured in RPMI 1640 (Gibco) containing 10% FBS (Gibco), 2 mM L-glutamine (BioShop), 10 mM HEPES (Roche), 1 mM sodium pyruvate (Sigma-Aldrich), 1 mM non-essential amino acids (Gibco), 55 μM β-mercaptoethanol (Gibco), 100 U/mL penicillin (Gibco), 100 μg/mL streptomycin (Gibco), 1.5 ng/mL rhL-2 and 10 ng/mL rhIL-7 (PeproTech, Cranbury, NJ). 16-24 hours later, activated T cells were transduced with lentivirus at an MOI of 1-2. T cells edited by CRISPR/Cas9 were washed with 1x PBS on day 3 to remove soluble activator, otherwise T cells were washed on day 4. Transduced cells were enriched with the EasySep Human CD271 Positive Selection Kit II (STEMCELL Technologies) on day 7-10 of culture. Culture yields were enumerated every 2-3d and supplemented with cytokine-containing media to dilute cultures to ~ 1.0×10^6^ cells/mL. Engineered T cell products were expanded for a total culture period of 14-15 days prior to use. In some cases, TAC T cell products were cryopreserved prior to use. In short, after culture cells were cryopreserved in CryoStor10 according to manufacturer’s directions at 20×10^6^ cells/mL (for downstream *in vitro* assays). Prior to use of cryopreserved TAC T cell products in any *in vitro* assay, cells were thawed according to manufacturer’s directions and rested for 24 hrs in cytokine-containing media (as above). Mean viability and recovery were 85.5% ( ± 7.2% std. dev.; 95% CI [83.2, 87.7]) and 57.2% ( ± 16.4% std. dev.; 95% CI [52.1, 62.3]), n=42.

All assays were performed in T cell medium without cytokines; FBS lots were assessed compared to previous lots to ensure similar T cell manufacturing (expansion, viability, cryopreservation) and functionality (proliferation, cytotoxicity, and cytokine production).

### CRISPR/Cas9 editing of T cells

T cell gene-editing was accomplished by electroporation of complexed gRNA/Cas9 ribonucleoprotein (RNP). To generate RNP, a triple sgRNA pool (Synthego, Redwood City, CA, USA) or negative control gRNA (IDT, Newark, NJ, USA) was complexed with 20 pmol Alt-R HiFi Cas9 V3 (IDT) at a 3:1 sgRNA , Cas9 molar ratio for 10-15 min at room temperature. Prior to electroporation activated T cells were pooled and washed with 1x PBS. 5x10^5^ T cells per electroporation were resuspended in buffer T (Thermo Fisher Scientific) and mixed with complexed RNP (20 pmol on a Cas9 basis) and shocked using a NEON electroporation system (Thermo Fisher Scientific) set to 1600 V, 10 ms pulse width, and 3 pulses. Immediately after electroporation, T cells were dispensed into antibiotic-free T cell medium containing rhIL-2 and rhIL-7. To edit the *TNFRSF8* gene, exon 6 was targeted with sgRNAs with sequences (5’ – 3’), AAATTACCTGGATCTGAACT, AGCTGCTTCTAAACTGACGA, and ACACCGGGGTGGGCTTGGCC. To edit the *FAS* gene, exon 2 was targeted with sgRNAs with sequence (5’ – 3’), CACUUGGGCAUUAACACUUU, UACAGUUGAGACUCAGAACU, and GUGUAACAUACCUGGAGGAC. Genomic DNA was collected from T cells on d14/15 with QuickExtract DNA extraction solution 1.0 (Lucigen, Hoddesdon, UK) following manufacturers instructions, and targeted exons were amplified by PCR and sequenced by sanger sequencing. Indel analysis was performed by Synthego ICE (45) with PCR/sequencing primers (5’ – 3’) for *TNFRSF8* exon 6, CTCCCCCTCATCTCAAGAGCTATC and TGAGCCTCAAACCAAAGCAAGA; *FAS* exon 2 TGAAGAACCTGAGATCCAAACTGCT and TGGTAGATCCTAATCAGTTTTGACATGA.

### Flow cytometry

Flow cytometry data were collected with BD LSR II (V/B/R or V/B/YG/R laser configuration), BD LSR Fortessa (V/B/R laser configuration) or Beckman Coulter CytoFLEX LX (NUV/V/YG/B/R laser configuration) and analyzed using FCS Express v7 Software (DeNovo Software, Pasadena, CA).

### Phenotypic characterization of T cell products

Surface expression of BCMA TAC constructs was determined by staining with recombinant human BCMA-Fc protein (R&D Systems, Minneapolis, MN), followed by goat anti-human IgG (to detect BCMA-Fc) and antibodies against markers CD4, CD8α, CD30, FAIM3, Fas, and/or NGFR. Antibody-fluorochrome combinations, cat #, and RRID# are indicated in the table following the materials section. Unless otherwise stated, all stains were done at room temperature for 30 minutes in PBS + 2% BSA + 2.5 mM EDTA. Staining was assessed by flow cytometry.

Transduction efficiency of TAC T cells was evaluated by co-expression of the TAC receptor and truncated NGFR in bulk, CD4, and/or CD8 populations (refer to **Supplemental Figure 8** for gating strategy). Knockout of Fas was similarly detected (refer to **Supplemental Figure 9**).

To assess expression of FAIM3 and CD30 of unedited TAC T cells in response to LCL161, 72-hour cell stimulation with BCMA-coated microbeads and LCL161 (5 μM) or vehicle was used. FAIM3 expression was abrogated in response to stimulation, whereas CD30 expression required stimulation (refer to **Supplemental Figure 10** for gating strategy). To assess expression of FAIM3 and CD30 on CD30-edited and/or FAIM3 overexpressing TAC T cells 72-hour stimulation with 1 μg/mL plate-bound agonistic anti-CD3 (clone, OKT3) and LCL161 (5 μM) or vehicle was utilized (refer to **Supplemental Figure 11** for gating strategy).

### *In vitro* cytotoxicity assay

For luciferase-based cytotoxicity assays TAC T cells were co-cultured in triplicate with 2.5x10^4^ luciferase-expressing RPMI 8226 or MM.1S cells in the presence of DMSO or LCL161 at indicated effector:target ratios for 48 hours at 37°C in opaque-white flat-bottom 96-well microplates. Following co-culture, 0.15 mg/mL D-Luciferin (Perkin Elmer, Waltham, MA) was added, and luminescence was measured with an open filter using a SpectraMax i3 (Molecular Devices, San Jose, CA) plate reader. Tumor cell viability was calculated as $\frac{\left(Emission-background\right)}{\left(Tumor\;alone-background\right)}\times 100\%$ . For flow-based cytotoxicity assays, engineered T cells were co-cultured with 2.5x10^4^ RPMI 8226 or MM.1S cells with DMSO or LCL161 at indicated effector:target ratios for 24 hrs at 37°C. Following co-culture, samples were stained for NGFR, CD138, and viability and CD138^+^NGFR^-^ cell viability was utilized as a measure of tumor cell survival.

### Microbead antigen loading

Protein G-conjugated ~6-7 μm polystyrene beads (Spherotech, Lake Forest, IL, USA) were coated with 50 ng BCMA-Fc chimera protein (R&D Systems, Minneapolis, MN, USA) per 10^6^ beads in PBS + 0.1% BSA at a concentration of 5x10^6^ beads/mL and incubated rotating overnight at 4°C. Immediately prior to use, beads were resuspended in T cell medium without cytokines to achieve the desired effector:target ratio.

### Proliferation assay

Engineered T cells were labelled with CellTrace Violet dye (Thermo Fisher Scientific) and stimulated with MM.1S tumor targets or antigen-coated microbeads at a 1:1 effector:target ratio (non-stimulated control wells were also plated to determine undivided peak for proliferation modeling). After 4 days at 37°C, co-cultures were stained with Live/Dead Fixable stain and antibodies against markers CD8α, CD4, NGFR, CD3, and/or CD138, and mixed with absolute counting beads (Thermo Fisher Scientific). Flow cytometry data were acquired as indicated above, refer to **Supplemental Figure 12** for gating strategy. Results were analysed using Proliferation Plots in FCS Express 7. In short, non-stimulated control T cells were used to identify and fix a Starting Generation within Proliferation Fit Options. Proliferation Fit Statistics were used to calculate the percent of original cells that divided (percent divided). Absolute cell yield was calculated as, $cell\;count=\;\frac{\#\;CD3+\;live\;events}{\#\;bead\;events}\times \#\;beads\;loaded\;into\;sample$ .

### Clariom-S RNA transcriptome analysis

5x10^5^ TAC T cells were stimulated with 0.05 μg/mL plate-bound BCMA-Fc antigen in a 24-well plate in the presence of 0.625 μM LCL161 or DMSO for 24 or 72 hrs. After stimulation, T cells were collected, washed with 1x PBS to remove media, then processed to collect total RNA. For the 24 hour stimulation, dead cells were removed using the Miltenyi Dead Cell removal kit. Briefly, T cells were homogenized by centrifugation through QIAshredder columns (QIAGEN, Hilden, Germany), and RNA was purified from the resultant lysates by RNeasy Mini Plus (QIAGEN) RNA purification kit as per manufacturer’s instructions. Purified RNA was assessed for quality above RIN>7 by NanoDrop OneC spectrophotometry (Thermo Fisher Scientific) and 2100 Bioanalyzer (Agilent, Santa Clara, CA, USA). Total RNA was hybridized using a Clariom-S Pico genechip (refer to GEO accession #GSE227986 for further details), and resultant data was analyzed using the Transcriptome Analysis Console (Thermo Fisher Scientific) to determine differential gene analysis (DGA) between DMSO and LCL161 group with donor as a repeated measure. Raw and normalized data is available through the NCBI Gene Expression Omnibus repository under accession #GSE227986. To generate volcano plots, the differentially expressed gene were input using the EnhancedVolcano() package in R (46). Genes were identified as significant with a p-value below 0.05 and a fold change >1.75/<-1.75. Gene ontology (GO) over-representation analysis was conducted using the clusterProfiler() package in R (47–49). Specifically, the biological processes (BP) ontology was used to identify enriched pathways. A Benjamin & Hochberg (50) (BH) correction was applied *post-hoc* and gene sets were identified as significant with an adjusted p-value below 0.05. All analyses were performed using R Statistical Software [v4.2.1 (49)].

### Western blotting

T cell NF-κB2 p100/p52 and XIAP analysis was accomplished by first stimulating 5x10^5^ TAC T cells on 0.05 μg/mL plate-bound BCMA-Fc antigen in a 24-well plate in the presence of 0.625 μM LCL161 or DMSO for 24, 48, or 72 hrs. After stimulation, T cells were collected, washed twice in 1x PBS, then frozen as cell pellets at -20°C. Protein lysates were generated from cell pellets by lysis with RIPA lysis buffer (Thermo Fisher Scientific) containing protease inhibitors (Roche, Basel, Switzerland) for 15 min on ice, followed by 15 min centrifugation at 15000 RCF 4°C. Lysates were quantified by BCA assay (Thermo Fisher Scientific), and 8.5 (NKFB2) or 10 (XIAP) μg protein was loaded onto a 4-20% gradient gel (Bio-Rad). Proteins were then transferred to a nitrocellulose membrane, stained for total protein by REVERT 700 total protein stian (Li-COR Biosciences, Lincoln, NE, USA), and then blotted for NF-κB2 (Cell Signaling Technologies) or XIAP (Cell Signaling Technologies). All imaging was accomplished using an Odyssey Lx Imaging System (Li-COR Biosciences) set to auto mode. Blots were analyzed using Empiria Software (Li-COR Biosciences) where NF-κB2 p100/p52, or XIAP, signals were normalized to total protein signal of their respective wells (refer to **Supplemental Figure 7** for an example). For analysis of tumor cells, MM.1S and RPMI 8226 cells were pelleted and lysed in an SDS-RIPA buffer (10 mM Tris-HCl, 150 mM NaCl, 10 mM KCl, 1 mM EDTA, 0.5% deoxycholic acid, 0.5% Tween 20, 0.5% NP-40, 0.1% SDS) with protease and phosphate inhibitor cocktail (MiliporeSigma) for 20 minutes on ice. Cells were then sonicated for 10 seconds at 20% amplitude before centrifugation and protein quantification by DC protein assay (Bio-Rad). Equal amount of protein (25 μg) was loaded onto 10% SDS-PAGE gels. Immunoblotting was performed overnight at 4°C with the primary antibodies, p100/p52 (Cell Signaling Technologies), NIK (Abcam, Cambridge, UK), β-Tubulin (DSHB, Iowa city, IA, USA), cIAP1/2 (MBL International, Woburn, MA, USA), and XIAP (MBL International). The next day, bound primary antibodies were probed with secondary antibodies conjugated to either Alexa Fluor680 (Thermo Fisher Scientific) or IRDye 800CW (Li-COR Biosciences) at room temperature for 1 hour, then blots were scanned on the Odyssey Infrared Imaging System (Li-COR Biosciences).

### T cell apoptosis and AICD assay

T cell apoptosis was investigated by stimulating 5x10^5^ engineered T cells 1:1 with antigen-loaded microbeads or MM.1S tumor cells for 24, 48, or 72 hrs in the presence of DMSO or LCL161 at indicated concentrations. Afterwards, cells were immediately stained with live/dead fixable viability dye at room temperature for 20 minutes, fixed with BD Cytofix, and stained for phosphatidylserine and CD3 surface expression on ice for 60 min. Cells were analyzed by flow cytometry and populations were defined as live (PS^-^ live/dead^-^), early apoptotic (PS^+^ live/dead^-^), and late apoptotic (PS^+^ live/dead^+^), refer to **Supplemental Figure 13** for gating strategy.

To investigate AICD in TAC T cells, cells were stimulated as above with antigen-loaded microbeads for 48 hrs in the presence of DMSO or LCL161. Cells were stained with live/dead fixable viability dye at RT, fixed with BD Cytofix, stained for phosphatidylserine on ice for 60 min, permeabilized with BD Phosflow perm/wash I as per manufacturers guidelines, then stained intracellularly for active caspase 3. Percent apoptotic in live cells was defined as live/dead^-^ PS^+^ active-caspse3^+^, refer to **Figure 6B** for gating strategy.

### Tumor cell TNF-sensitivity assay

2x10^5^ MM.1S or RPMI 8226 cells were incubated with 5 μM LCL161 or DMSO and in the presence of rhTNFα (BD Biosciences) at indicated concentrations for 48 hrs. After incubation, cells were collected and stained using a fixable live/dead dye. Samples were analyzed by flow cytometry to determine viability.

### Statistics

All statistical analyses were performed in GraphPad Prism version 9.4 for Windows (San Diego, CA). Paired two tailed t tests, or multiple paired t tests using the Holm-Šídák method, were utilized to compare between two samples. An ordinary one-way ANOVA using the Dunnett multiple comparison test was used to compare the means of three or more unmatched groups. * = p > 0.05, ** = p > 0.01, *** = p > 0.001, and N.S. = not significant.TABLE

### Reagents

**Table:** 

**Reagent**	**Source**	**Identifier**
Goat anti-human IgG (H+L)-PE	Jackson ImmunoResearch	Cat# 109-115-098, RRID , AB_2337675
Anti-human CD4-Pacific Blue	BD Biosciences	Cat# 558116, RRID , AB_397037
Anti-human CD4-APC-H7	BD Biosciences	Cat# 560158, RRID , AB_1645478
Anti-human CD4-AlexaFluor 700	Thermo Fisher Scientific	Cat# 56-0048-82, RRID , AB_657741
Anti-human CD8α-AlexaFluor 700	Thermo Fisher Scientific	Cat# 56-0086-82, RRID , AB_657756
Anti-human CD8α-PerCP-Cyanine5.5	Thermo Fisher Scientific	Cat# 45-0088-42, RRID , AB_1582255
Anti-human CD30-PE	Miltenyi Biotec	Cat# 130-120-783, RRID , AB_2784118
Anti-human FAIM3-APC	BioLegend	Cat# 398103, RRID , AB_2876722
Anti-human NGFR-VioBright FITC	Miltenyi Biotec	Cat# 130-104-893, RRID , AB_2661084 Cat# 130-113-985, RRID , AB_2734063
Anti-human NGFR-Brilliant Violet 421	BD Biosciences	Cat# 562562, RRID , AB_2737657
Anti-human CD138-APC	BioLegend	Cat# 356505, RRID , AB_2561879
Anti-human CD3-Brilliant Violet 605	BioLegend	Cat# 300460, RRID , AB_2564380
Anti-human FasR-APC	BioLegend	Cat# 305612, RRID , AB_314550
Functional grade anti-human CD3	Thermo Fisher Scientific	Cat# 16-0037-85, RRID , AB_468855
Anti-NF-κB2 P100/p52	Cell Signaling Technologies	Cat# 4882, RRID , AB_10695537
Anti-human NIK	Abcam	Cat# ab7204, RRID , AB_2139765
Anti-cIAP1/2	MBL International	Cat# CY-P1041, RRID , AB_10950764
Anti-XIAP	MBL International	Cat# M044-3, RRID , AB_592998
Anti-XIAP	Cell Signaling Technologies	Cat# 2045, RRID , AB_2214866
Anti-β-Tubulin	Developmental Studies Hybridoma Bank	Cat# E7, RRID , AB_528499
IRDye 800CW goat anti-rabbit	Li-COR Biosciences	Cat# 926-32211, RRID , AB_621843
Anti-human FasL-PE	BD Biosciences	Cat# 564261, RRID , AB_2738713
Anti-phosphatidylserine-AlexaFluor 488	MilliporeSigma	Cat# 16-256, RRID , AB_492616
Anti-human active caspase 3-Alexa Fluor 647	BD Bioscience	Cat# 560626, RRID , AB_1727414
LIVE/DEAD fixable Near-IR dead cell stain	Thermo Fisher Scientific	Cat# L10119
Cytofix buffer	BD Biosciences	Cat# 554655
Phosflow perm/wash I	BD Bioscience	Cat# 557885
123count eBeads	Thermo Fisher Scientific	Cat# 01-1234-42
Protein G-conjugated ~6 μm polystyrene beads	Spherotech	Cat# PGP-60-5
Alt-R S.p. HiFi Cas9 Nuclease V3	Integrated DNA Technologies	Cat# 1081061
Pierce RIPA Lysis buffer	Thermo Fisher Scientific	Cat# 89901
cOmplete, Mini, EDTA-free protease inhibitor tablets	Roche	Cat# 04 693 159 001
Protease and Phosphatase Inhibitor Cocktail	MilliporeSigma	Cat# PPC1010
DC Protein Assay Kit	Bio-Rad	Cat# 5000111
Pierce BCA assay	Thermo Fisher Scientific	Cat# 23227
4-20% TGX pre-cast gel, 15 lanes	Bio-Rad	Cat# 4561096
0.2 μm nitrocellulose membrane	Bio-Rad	Cat# 1620112
REVERT 700 Total protein stain kit	Li-COR Biosciences	Cat# 926-11010
Intercept blocking buffer (TBS)	Li-COR Biosciences	Cat# 927-60001
Intercept Antibody Diluent (T20 TBS)	Li-COR Biosciences	Cat# 927-65001
CellTrace Violet	Thermo Fisher Scientific	Cat# C34557
D-Luciferin	Perkin Elmer	Cat# 122799
rhBCMA-Fc chimera	R&D Systems	Cat# 193-BC
rhTNFα	BD Biosciences	Cat# 554618
LCL161	Novartis	N/A

## Data availability statement

The data presented in the study are deposited in the NCBI Gene Expression Omnibus repository, accession number GSE227986, released: https://www.ncbi.nlm.nih.gov/geo/query/acc.cgi?acc=GSE227986.

## Ethics statement

The studies involving human participants were reviewed and approved by Hamilton Integrated Research Ethics Board. The patients/participants provided their written informed consent to participate in this study. The animal study was reviewed and approved by McMaster Animal Research Ethics Board.

## Author contributions

AA, SB, EL, and JB conceptualized these studies. AA and JB designed experiments and interpreted results. AA, CS, AM, CG, CB, MS-J, and KB generated these data and performed analyses. AA and JB wrote this manuscript. SB, EL, and CG edited the manuscript. All authors contributed to the article and approved the submitted version.
