# Supplementary material for: LCL161 enhances expansion and survival of engineered anti-tumor T cells but is restricted by death signaling
Source: Front Immunol. 2023 Apr 17;14:1179827. doi: 10.3389/fimmu.2023.1179827 (PMC10150108; doi:10.3389/fimmu.2023.1179827)
Supplement: Supplementary file 1 [file DataSheet_1.docx]

Supplementary Material

LCL161 enhances expansion and survival of engineered anti-tumor T cells but is restricted by death signaling

Arya Afsahi, Christopher M Silvestri, Allyson E Moore, Carly F Graham, Kaylyn Bacchiochi, Martine St-Jean, Christopher L Baker, Robert G Korneluk, Shawn T Beug, Eric C LaCasse, and Jonathan L Bramson*

*** Correspondence:** Jonathan Bramson:
bramsonj@mcmaster.ca

# Supplementary Figures and Tables

## Supplementary Figures


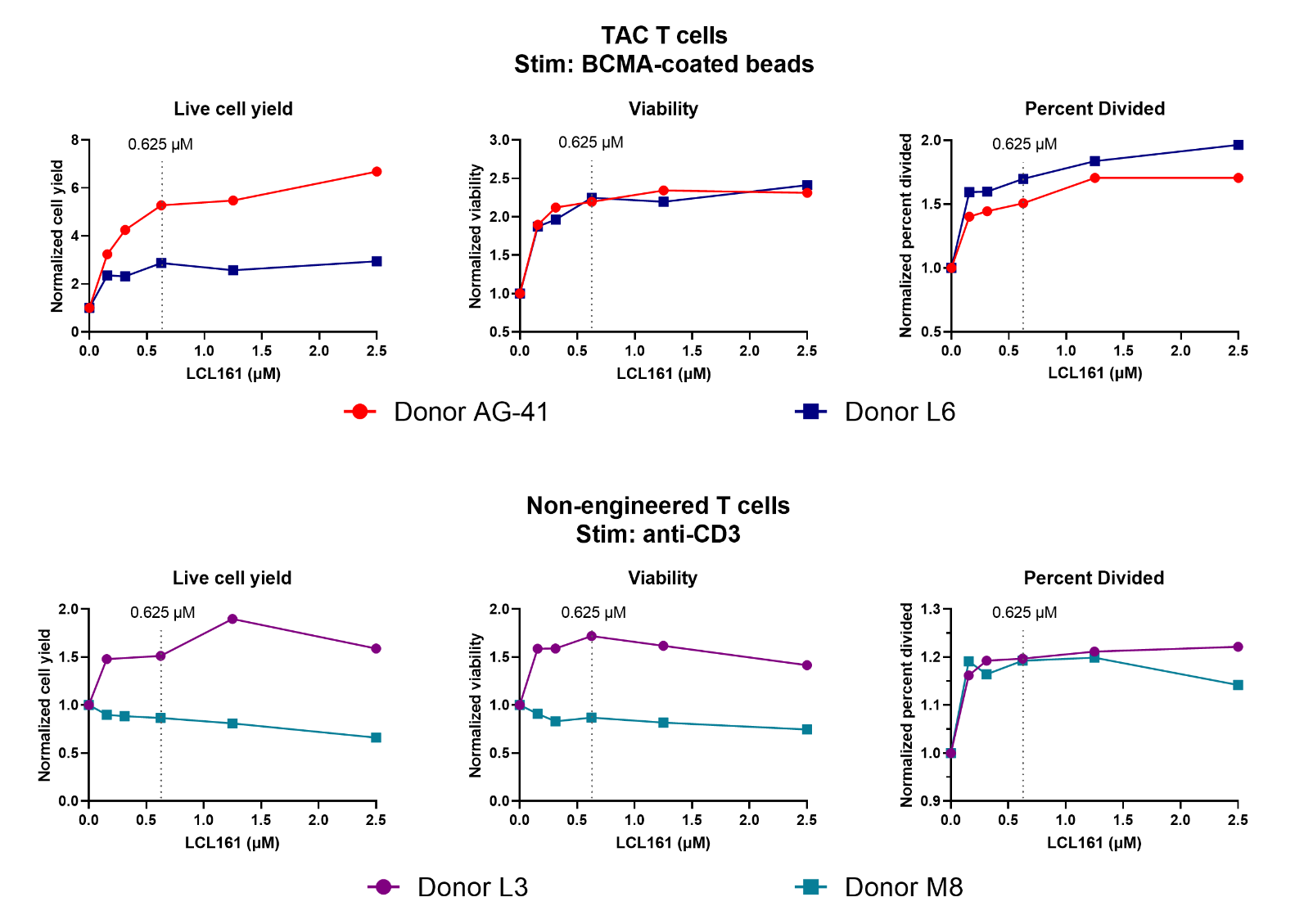


**Supplemental Figure 1. LCL161 enhancement of T cell proliferation plateaus at ~0.5 - 1 μM**. 0.5x10^6^ CellTrace Violet-labeled TAC T cells generated from 4 donors were stimulated in the presence of increasing concentration of LCL161 for 96 hrs with either BCMA-coated microbeads or 1 μg/mL plate-bound agonistic anti-CD3 antibody. Cells were then collected and analyzed by flow cytometry and proliferation statistics were modeled using FCS Express software. These data represent 2 independent experiments.


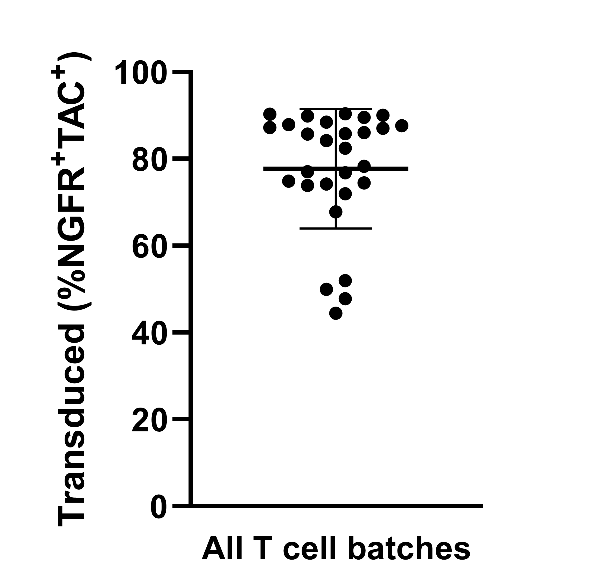


**Supplemental Figure 2. Overall transduction of engineered T cell batches.** TAC T cells generated from healthy or myeloma patient donors were phenotyped for expression of the TAC receptor and tNGFR transduction marker. Data comprises 8 healthy donors and 3 myeloma patient donors. Cells were collected and stained for flow cytometric analysis either post manufacturing or post-thaw from cryopreservation. n=28.


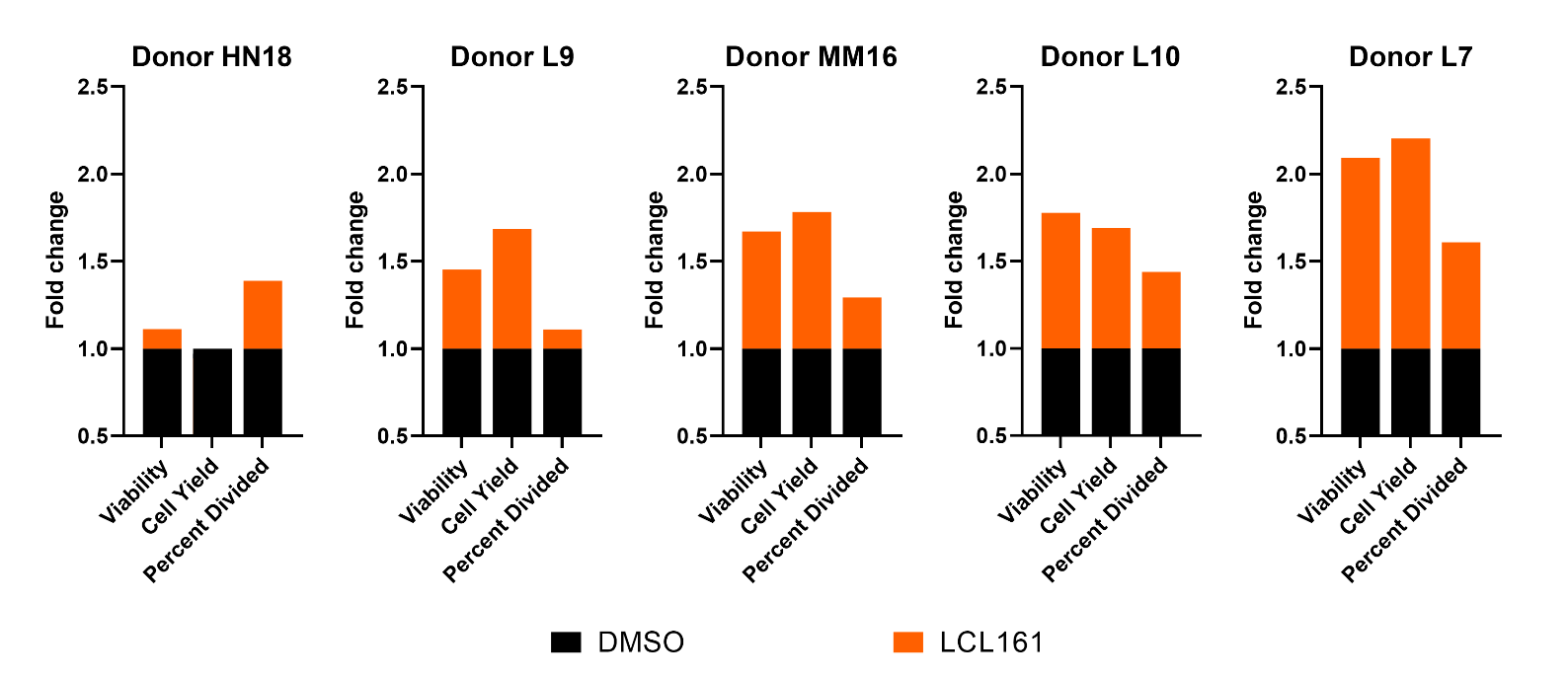


**Supplemental Figure 3. Proliferation metrics in response to stimulation with antigen and LCL161 amongst donors.** Protein G polystyrene beads were loaded with 50 ng BCMA-Fc/million beads overnight at 4°C while mixing. CTV-labelled TAC T cells were cultured with loaded beads at an E:T of 1:1 for 96 hrs. LCL161 was utilized at a concentration of 0.625 µM. T cells were harvested and stained for flow cytometric analysis. These data represent 3 independent experiments.


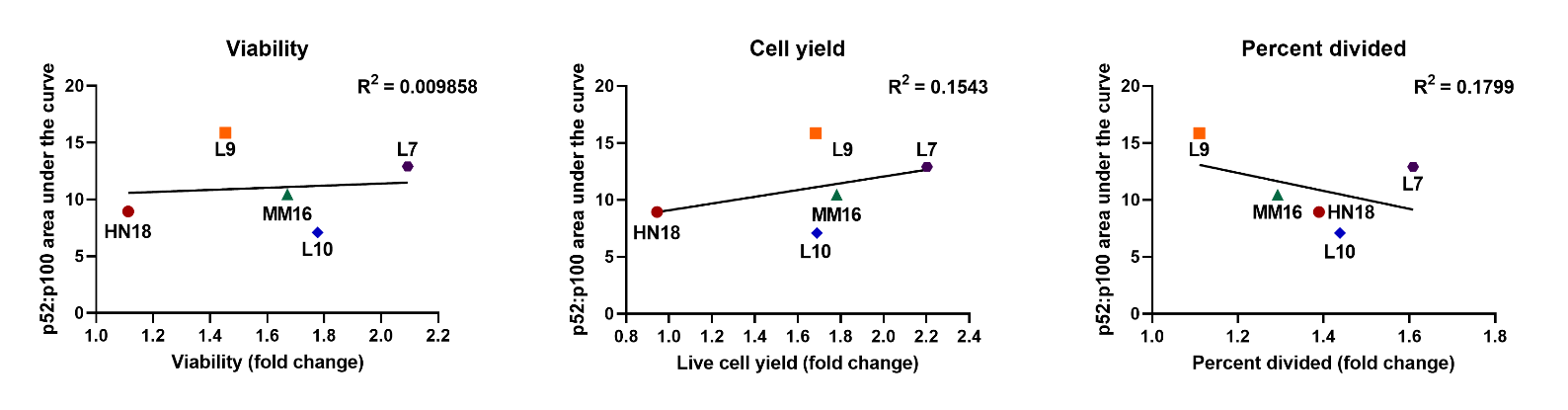


**Supplemental Figure 4.** **There is no relationship between individual proliferation parameters to p52:p100 ratio area-under-the-curve.** Area-under-the-curve values of the p52:p100 curves in Figure 1C were compared to the individual proliferation parameters calculated in Supplemental Figure 3.


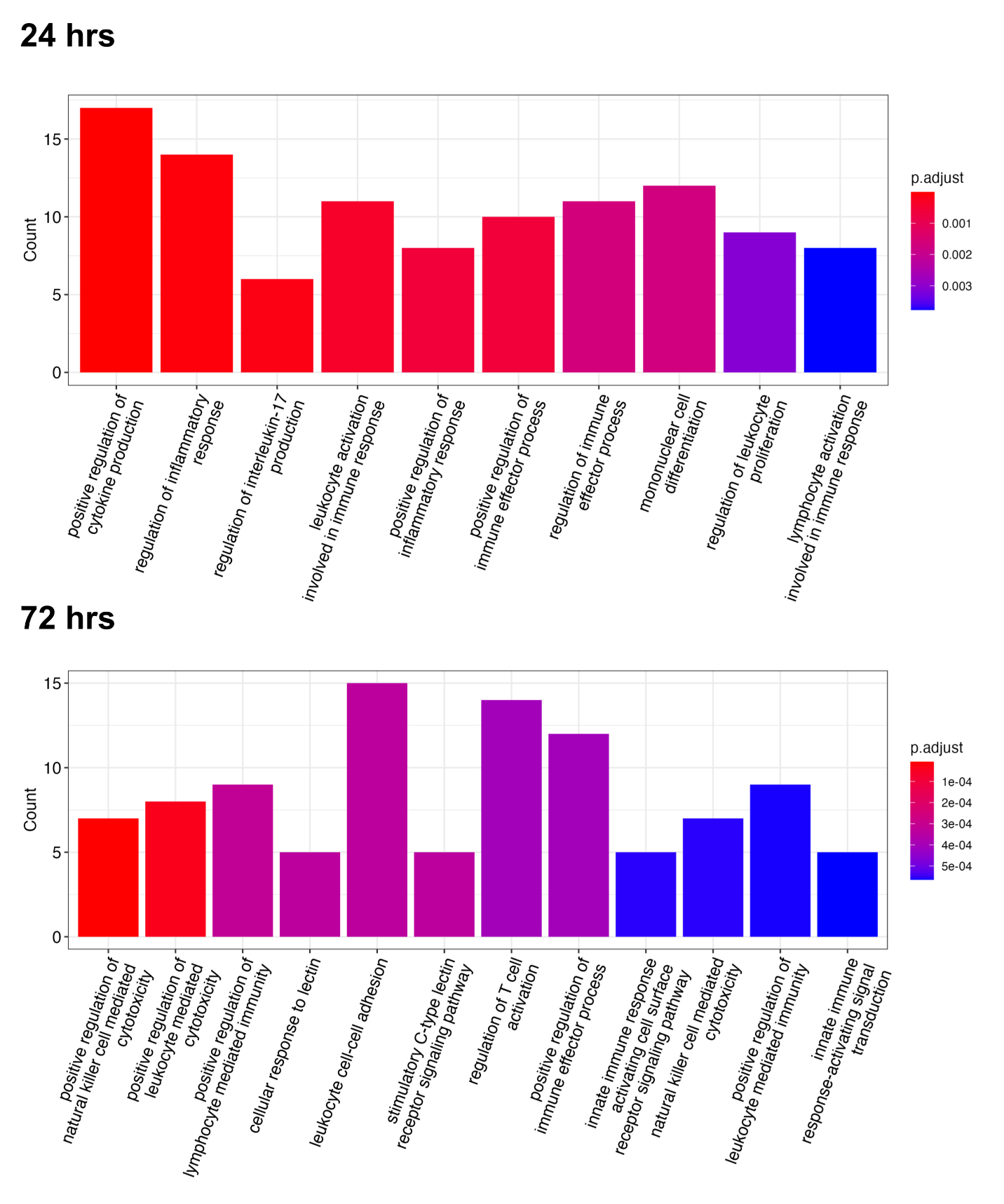


**Supplemental Figure 5. Gene-signature enrichment analysis of LCL161-differentially expressed genes.** Bar plots were generated from the results of an over-representation analysis using the gene ontology (GO) database with the 24 and 72 hour results. The GO analysis was done using the biological processes (BP) ontology with the Benjamin Hochberg (FDR) p-value adjustment. An adjusted p-value threshold of 0.01 was used to identify significant GO terms.


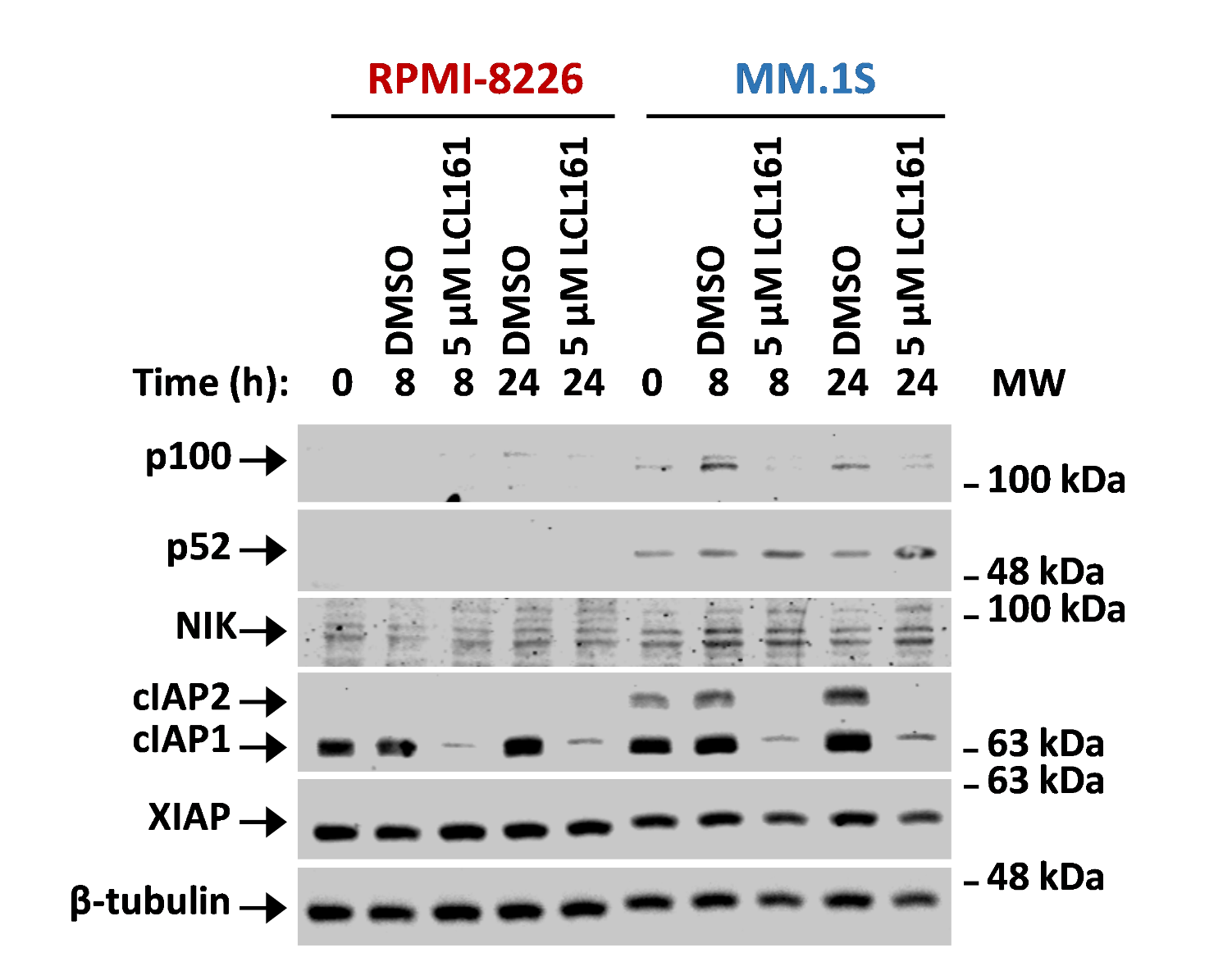


**Supplemental Figure 6. Expression of cIAP1/2 and ncNF-κB signaling proteins in the multiple myeloma cell lines RPMI 8226 and MM.1S**. Cell lines were cultured from thaw for 1 week prior to analysis. Cells were pelleted and cellular lysates were utilized for western blot analysis.


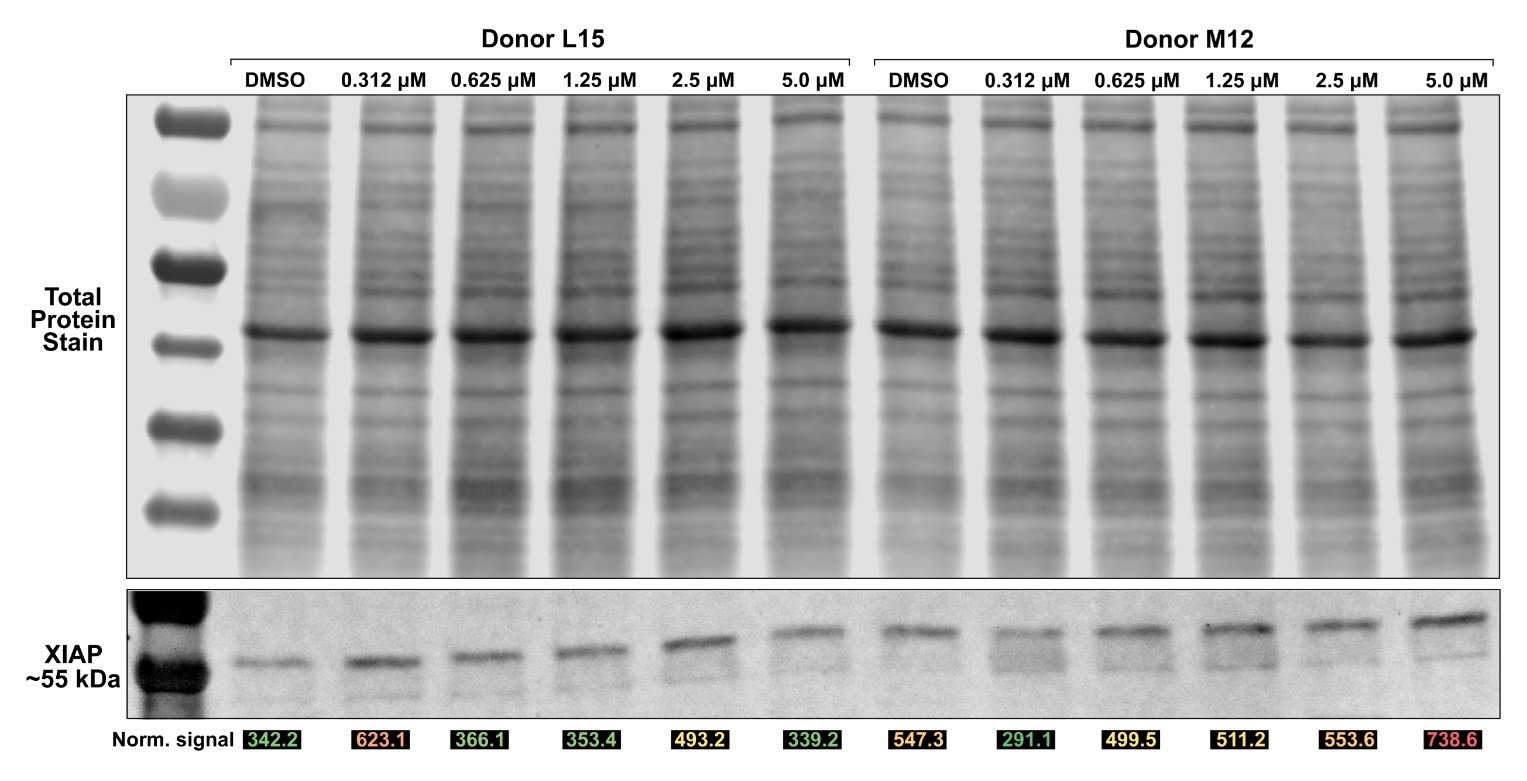


**Supplemental Figure 7. Measurement of XIAP protein levels in TAC T cells stimulated with antigen-alone in the presence of LCL161.** TAC T cells were stimulated for 72 hrs with plate-bound BCMA with increasing concentration of LCL161. After stimulation, cells were collected, fractured and processed for protein lysates. 12.5 μg of protein was loaded onto a 4-20% TGX polyacrylamide gel with denaturing conditions and transferred to a nitrocellulose membrane. Total protein signal was measured by Li-Cor REVERT stain for normalization and then washed prior to immunoblotting for XIAP 1:1000 (Cell Signaling Technologies). Normalized XIAP signal was calculated using Li-Cor Empiria software.


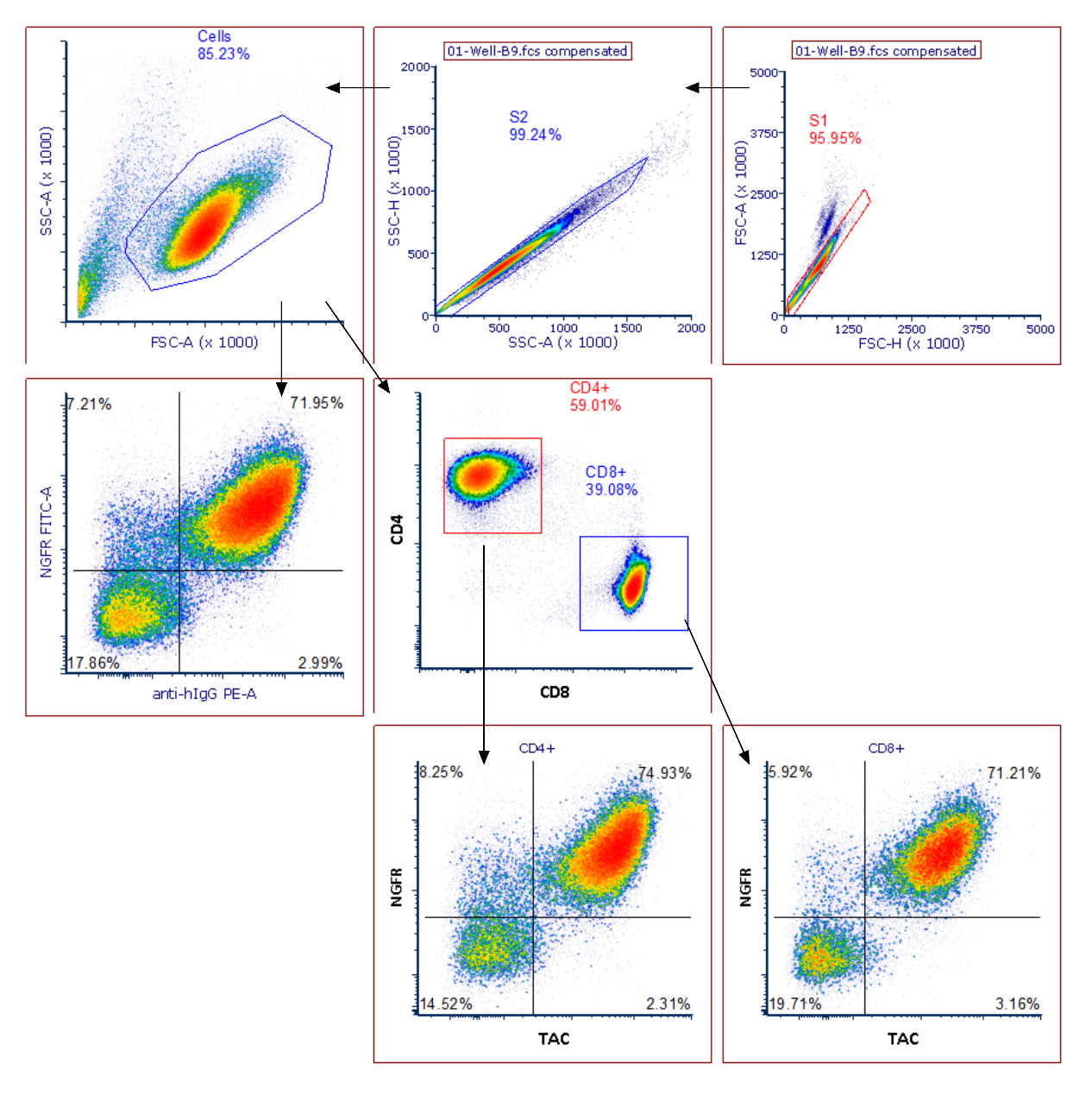


**Supplemental Figure 8. Flow cytometry gating strategy for phenotypic characterization of T cells transduced to express the BCMA-specific TAC and a truncated NGFR.** T cells were stained after manufacturing with rhBCMA-Fc to label TAC+ T cells, then subsequently stained with fluorochrome-conjugated antibodies specific to hIgG, CD4, CD8, and NGFR. Cells were gated as singlets (FSC) > singlets (SSC) > lymphocytes > bulk transduced (TAC+NGFR+) T cells; or CD4/CD8 > transduced (TAC+NGFR+) CD4+ or CD8+ T cells.


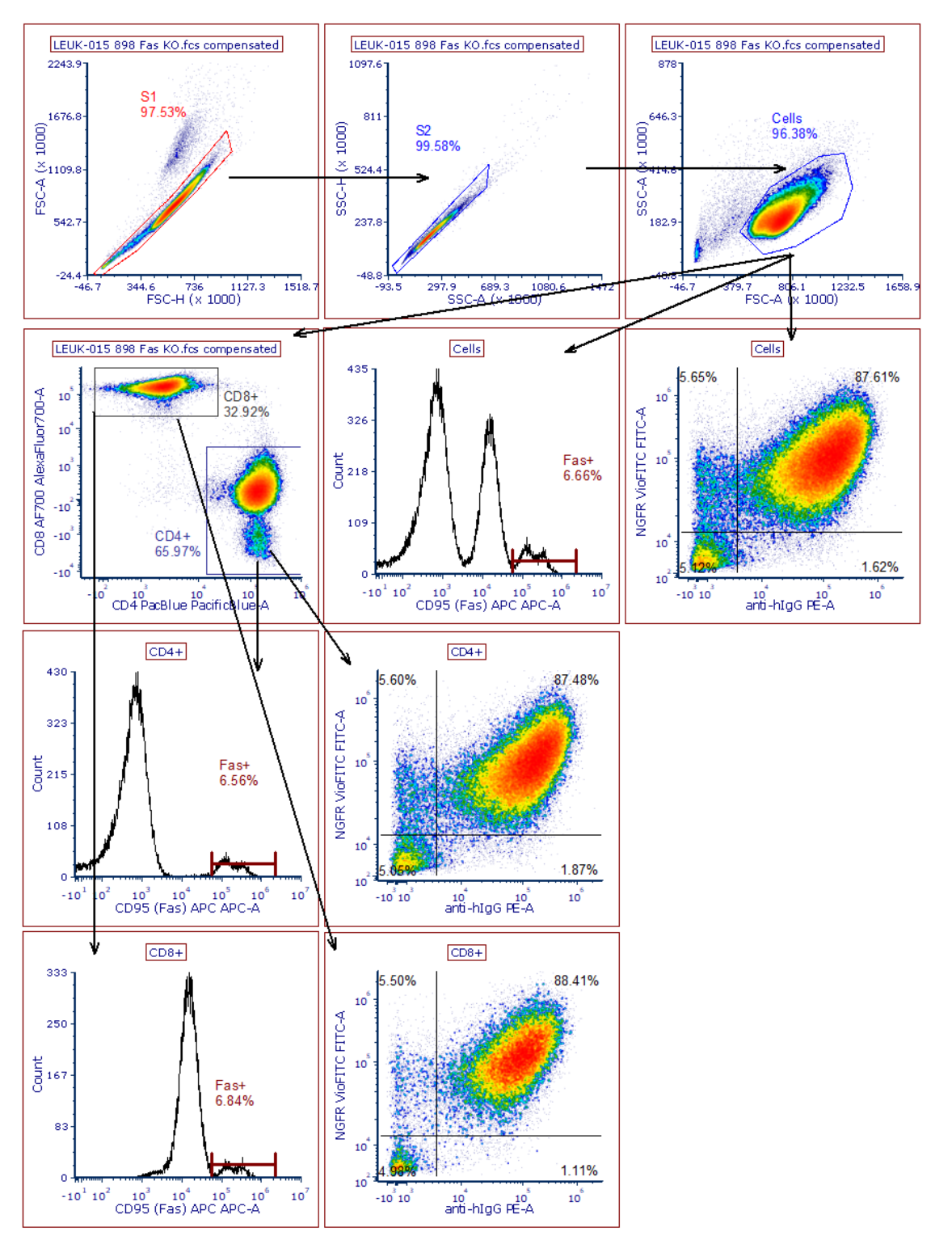


**Supplemental Figure 9. Flow cytometry gating strategy for phenotypic characterization of Fas-edited TAC T cells.** TAC T cells were stained after manufacturing with rhBCMA-Fc to label TAC+ T cells, then subsequently stained with fluorochrome-conjugated antibodies specific to hIgG, CD4, CD8, NGFR, and Fas. Cells were gated as singlets (FSC) > singlets (SSC) > lymphocytes > bulk transduced (TAC+NGFR+) T cells and Fas-/+; or CD4/CD8 > transduced (TAC+NGFR+) CD4+ or CD8+ T cells and Fas-/+.


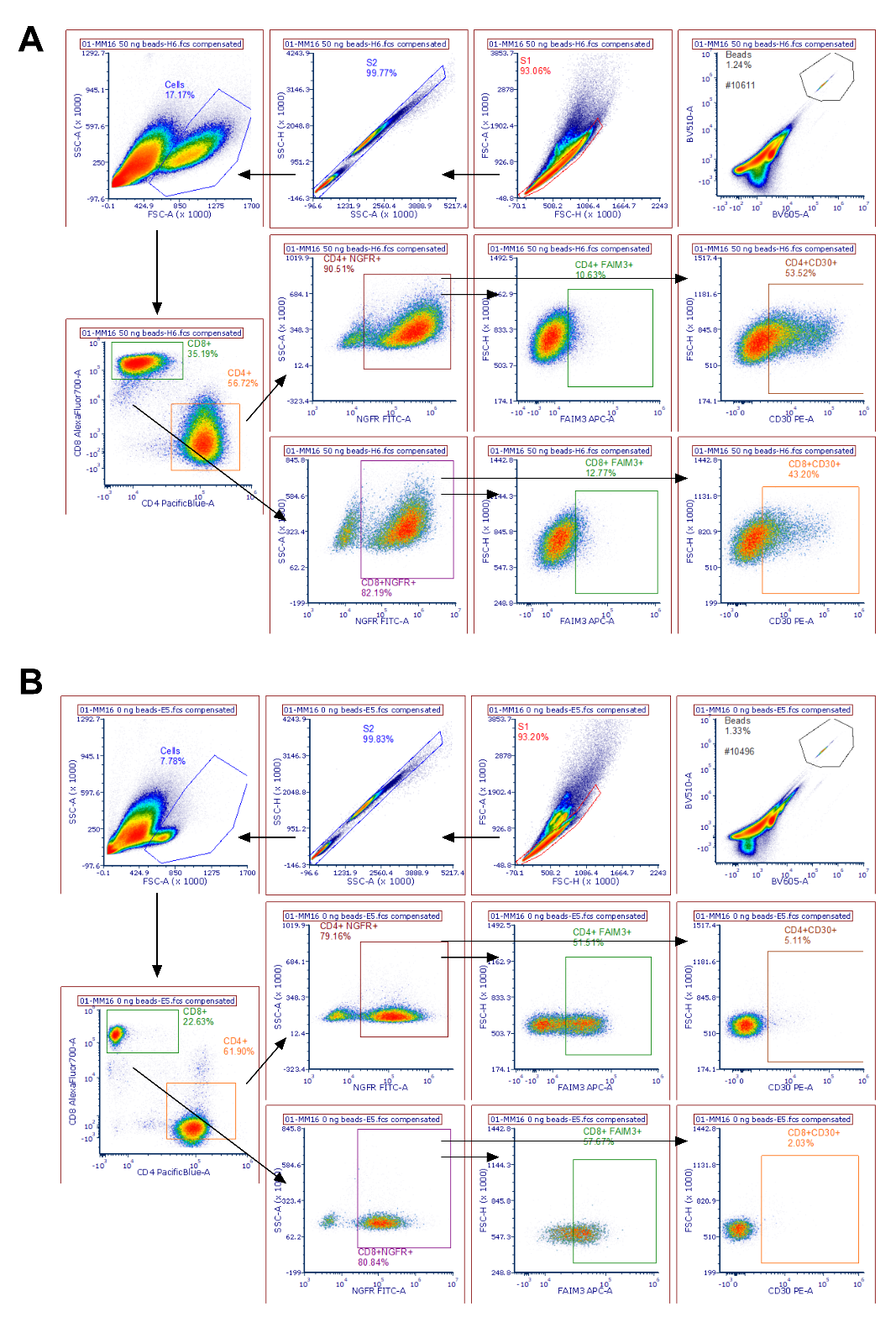


**Supplemental Figure 10. Flow cytometry gating strategy for phenotypic characterization of CD30 and FAIM3 in unedited TAC T cells.** An example plot is shown of TAC T cells that were stimulated with **(A)** BCMA-coated microbeads and LCL161 or **(B)** empty beads and vehicle. T cells were stained with fluorochrome-conjugated antibodies specific to CD4, CD8, NGFR, CD30, and FAIM3. 123eBead counting beads were included. Cells were gated as singlets (FSC) > singlets (SSC) > lymphocytes > CD4/CD8 > transduced (NGFR+) CD4+ or CD8+ T cells > CD30-/+ or FAIM3-/+.


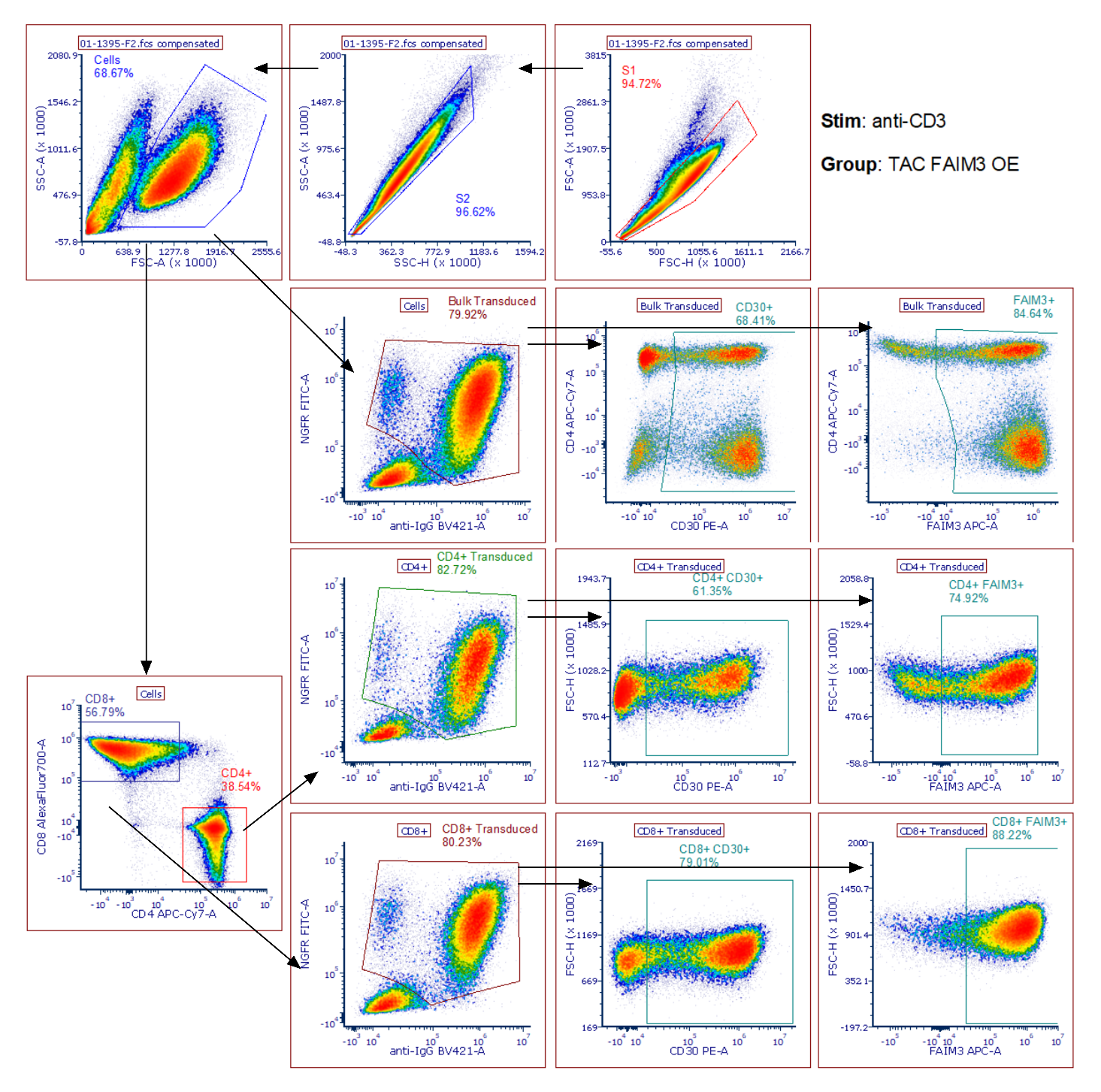


**Supplemental Figure 11. Flow cytometry gating strategy for phenotypic characterization of CD30 and FAIM3 in CD30-edited and/or FAIM3 overexpressing TAC T cells.** An example plot is shown of FAIM3 OE TAC T cells that were stimulated with 1 μg/mL plate-bound agonistic anti-CD3. T cells were stained with rhBCMA-Fc to label TAC+ T cells, then subsequently stained with fluorochrome-conjugated antibodies specific to hIgG, CD4, CD8, NGFR, CD30, and FAIM3. Cells were gated as singlets (FSC) > singlets (SSC) > lymphocytes > bulk transduced (TAC+NGFR+); or CD4/CD8 > bulk transduced CD30-/+ or FAIM3-/+; or CD4+ transduced CD30-/+ or FAIM3-/+; or CD8+ transduced CD30-/+ or FAIM3-/+.


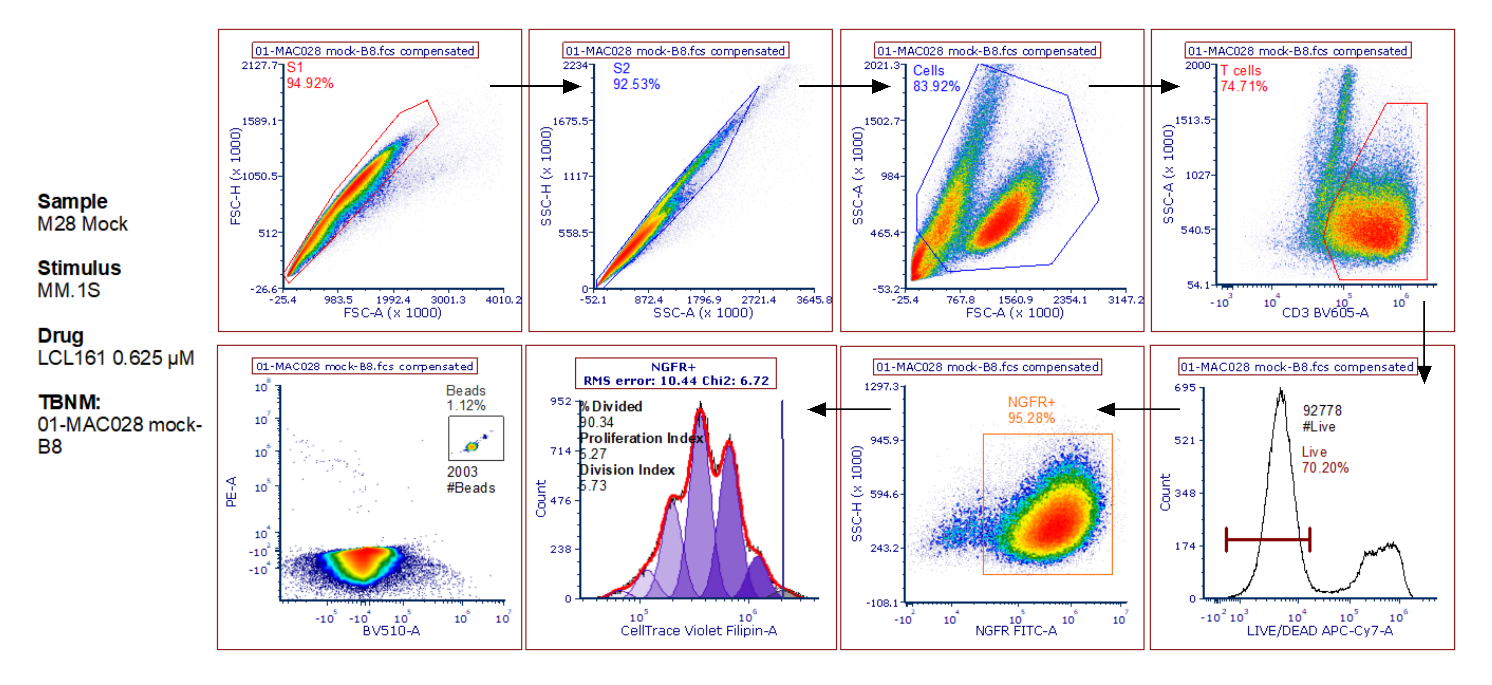


**Supplemental Figure 12. Flow cytometry gating strategy for CellTrace Violet dye-dilution proliferation assay.** An example plot is shown of CellTrace Violet-labeled TAC T cells that were stimulated 1:1 with MM.1S myeloma cells in the presence of LCL161. After stimulation T cells were stained with fixable viability dye and then fluorochrome-conjugated antibodies specific to CD3, CD4, CD8, and NGFR. 123eBead counting beads were included to enumerate total cells. Cells were gated as singlets (FSC) > singlets (SSC) > total cells > CD3+ > live cells > NGFR+ > proliferation modeling.


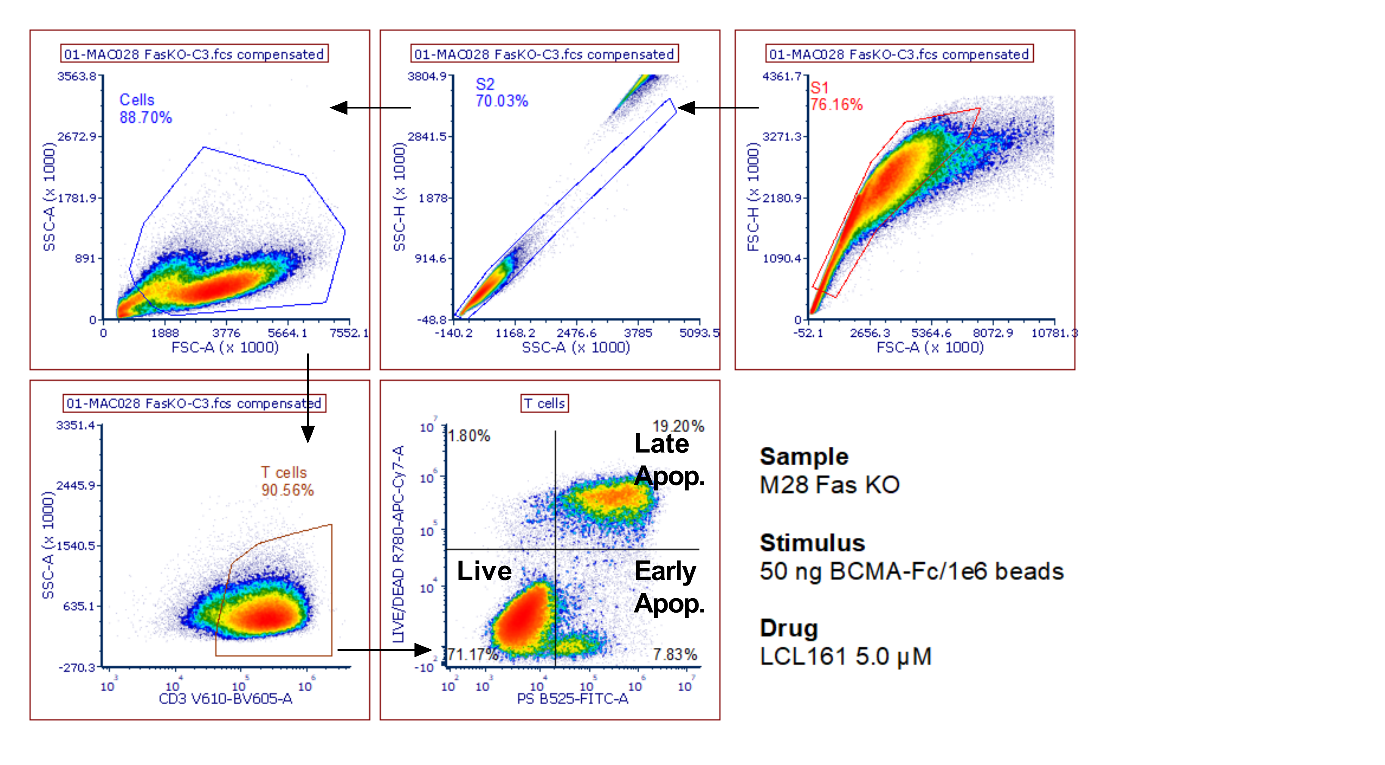


**Supplemental Figure 13. Flow cytometry gating strategy for T cell apoptosis assay.** An example plot is shown of TAC T cells that were stimulated 1:1 with antigen-loaded microbeads in the presence of LCL161 for 48 hrs. After stimulation T cells were stained with fixable viability dye for 20 min at room temperature, and then stained with fluorochrome-conjugated antibodies specific to CD3 and phosphatidylserine on ice for 1 hour. Cells were gated as singlets (FSC) > singlets (SSC) > total cells > CD3+ > viability versus phosphatidylserine. Live cells were gated as viability dye- and phosphatidylserine-. Early apoptotic were gated as viability dye- and phosphatidylserine+. Late apoptotic were gated as viability dye+ and phosphatidylserine+.

## Supplementary Tables

**Supplemental Table 1. Patient donor multiple myeloma staging.** Cancer staging of multiple myeloma donors used for TAC T cell manufacturing at time of PBMC donation.

| Donor | Multiple Myeloma staging |
| --- | --- |
| MM-16 | 3 |
| HN-18 | 1 |
| AG-41 | 1 |
